# MtMTP2-Facilitated Zinc Transport Into Intracellular Compartments Is Essential for Nodule Development in *Medicago truncatula*

**DOI:** 10.3389/fpls.2018.00990

**Published:** 2018-07-10

**Authors:** Javier León-Mediavilla, Marta Senovilla, Jesús Montiel, Patricia Gil-Díez, Ángela Saez, Igor S. Kryvoruchko, María Reguera, Michael K. Udvardi, Juan Imperial, Manuel González-Guerrero

**Affiliations:** ^1^Centro de Biotecnología y Genómica de Plantas (UPM-INIA), Universidad Politécnica de Madrid, Madrid, Spain; ^2^Noble Research Institute, Ardmore, OK, United States; ^3^Instituto de Ciencias Ambientales, Consejo Superior de Investigaciones Científicas, Madrid, Spain; ^4^Escuela Técnica Superior de Ingeniería Agronómica, Alimentaría y de Biosistemas, Universidad Politécnica de Madrid (UPM), Madrid, Spain

**Keywords:** zinc, cation diffusion facilitator, metal transport protein, symbiotic nitrogen fixation, metal nutrition, nodulation

## Abstract

Zinc (Zn) is an essential nutrient for plants that is involved in almost every biological process. This includes symbiotic nitrogen fixation, a process carried out by endosymbiotic bacteria (rhizobia) living within differentiated plant cells of legume root nodules. Zn transport in nodules involves delivery from the root, via the vasculature, release into the apoplast and uptake into nodule cells. Once in the cytosol, Zn can be used directly by cytosolic proteins or delivered into organelles, including symbiosomes of infected cells, by Zn efflux transporters. *Medicago truncatula* MtMTP2 (*Medtr4g064893*) is a nodule-induced Zn-efflux protein that was localized to an intracellular compartment in root epidermal and endodermal cells, as well as in nodule cells. Although the *MtMTP2* gene is expressed in roots, shoots, and nodules, *mtp2* mutants exhibited growth defects only under symbiotic, nitrogen-fixing conditions. Loss of MtMTP2 function resulted in altered nodule development, defects in bacteroid differentiation, and severe reduction of nitrogenase activity. The results presented here support a role of MtMTP2 in intracellular compartmentation of Zn, which is required for effective symbiotic nitrogen fixation in *M. truncatula.*

## Introduction

Zinc (Zn) is an essential nutrient for plants as a cofactor of enzymes or as a structural element (Coleman, 1998; Broadley et al., 2007). Consequently, plants grown in soils with low Zn bioavailability, which include some of the main agricultural areas of the world, have severe growth defects (Alloway, 2008; Marschner, 2012). These include interveinal chlorosis, necrotic leaves, and stunted growth, the result of alterations in the plethora of processes mediated by Zn proteins (Coleman, 1998; Broadley et al., 2007). To prevent this and ensure proper Zn allocation, plants have developed a complex network of transcription factors, transporters, and small Zn-chelating molecules that direct this metal to the proper tissue, cell compartments, and apoproteins (Assunção et al., 2010; Sinclair and Krämer, 2012; Olsen and Palmgren, 2014).

Zinc transport is mediated typically by four different transporter families: the Zrt1/Irt1-like (ZIP) and the yellow stripe-like (YSL) families for transport into the cytosol; and the metal tolerance protein (MTP) and Zn^2+^-ATPase families for efflux out of the cytosol (DiDonato et al., 2004; Eren and Argüello, 2004; Desbrosses-Fonrouge et al., 2005; Ishimaru et al., 2005; Olsen and Palmgren, 2014). Uptake of Zn from soils in dicots is mediated by ZIP proteins (Korshunova et al., 1999), in a process induced by soil acidification (Pedas and Husted, 2009). Zn can move symplastically from cell to cell and is released from endodermal cells into the xylem, via Zn^2+^-ATPases (Hussain et al., 2004). YSL transporters are likely candidates to mediate Zn loading into the phloem, as a Zn-nicotianamine complex (Waters et al., 2006). Within cells, Zn is transported into organelles by MTP or Zn^2+^-ATPases, either to be stored when in excess, or to be used to assemble Zn-proteins (Blaudez et al., 2003; Desbrosses-Fonrouge et al., 2005; Morel et al., 2009). Overall, these processes are regulated by a set of transcription factors that orchestrate Zn homeostasis (Assunção et al., 2010).

While leaves are the main Zn sink in most plants during vegetative growth (Broadley et al., 2007), legumes have an additional one: nitrogen-fixing root nodules (González-Guerrero et al., 2014, 2016). Nodules are root- or stem-associated organs that develop as a result of complex chemical exchanges with soil bacteria known as rhizobia (Downie, 2014). After detection of specific nodulation factors synthesized by the colonizing rhizobia (Oldroyd, 2013), cells of the root pericycle and cortex proliferate to originate the nodule primordia (Xiao et al., 2014). As the nodule develops, a root hair curls to surround the rhizobia in the root rhizoplane. The plasma membrane of this hair cell retracts into the cytosol, forming an infection thread that guides the rhizobia from the epidermis into the nodule core (Gage, 2002). There, in an endocytic-like process, rhizobia are released into the cytosol of cortical cells (Limpens et al., 2009). Under the proper physico-chemical conditions, rhizobia differentiate into bacteroids (Kereszt et al., 2011). Surrounded by a specialized plant-derived membrane, the symbiosome membrane, and protected from oxygen, bacteroids are able to synthesize nitrogenase, the iron-molybdenum enzyme complex responsible for converting atmospheric N_2_ into NH_4_^+^ (Rubio and Ludden, 2005). Fixed nitrogen is transferred to the host plant, in exchange for photosynthate and mineral nutrients from the plant (Udvardi and Poole, 2013). Two morphological types of nodules are known as follows: determinate and indeterminate (Brewin, 1991). Indeterminate nodules, such as those present in the genera *Medicago* or *Pisum*, are characterized by the presence of a persistent apical meristem(s) that produce cylindrical or coralloid-shaped organs (Vasse et al., 1990). As a consequence, different developmental zones can be distinguished in such nodules: the meristem or Zone I, the region where rhizobia colonize the nodule and differentiate into bacteroids or Zone II, the nitrogen fixation zone or Zone III, and, in old nodules, the senescent zone or Zone IV (Vasse et al., 1990). To this, some authors add an Interzone between Zones II and III, were oxygen levels transition from atmospheric levels (20%) to microaerobiosis (<1%); and a Zone V, where the rhizobia grow saprophytically (Timmers et al., 2000; Roux et al., 2014). In addition to the zonation pattern, determinate and indeterminate nodules differ in the process of bacteroid differentiation, that is irreversible for the majority of indeterminate nodules, to the extent that they cannot proliferate if released from the nodules. These bacteroids also have a higher ploidy level and larger size, as a consequence of cysteine-rich peptides released by the host cell that participate in bacteroid development (Van de Velde et al., 2010; Kereszt et al., 2011; Kondorosi et al., 2013; Stonoha-Arther and Wang, 2018).

The specific role(s) of Zn in symbiotic nitrogen fixation is not clear. Given the multitude of Zn-proteins in a cell, Zn could act at several different levels. It has been reported that plants growing under Zn deficiency suffer reduced growth and reduced nitrogenase activity (Ibrikci and Moraghan, 1993; O’Hara, 2001). Similar effects result from silencing the expression of *MtZIP6*, a *Medicago truncatula* Zn transporter located in the plasma membrane of nitrogen-fixing nodule cells (Abreu et al., 2017). Nodule development in these silenced plants was also affected, resulting in smaller nodules compared to wild-type controls. Understanding how reduced uptake of Zn into cells affects nodule development and symbiotic nitrogen fixation is complicated by the fact that Zn plays a role in numerous intracellular processes. Knowledge of the intracellular fate of Zn would help in this regard. To this end, we are characterizing Zn transporters likely to be involved in organelle loading, especially be members of the MTP and Zn^2+^-ATPase families.

Exploration of publicly available transcriptome databases (Benedito et al., 2008; Roux et al., 2014) revealed no Zn^2+^-ATPase to be upregulated during nodule development. In contrast, *M. truncatula MtMTP2* (*Medtr4g064893*) was found to be expressed at higher levels in nodules than in any other organ. Here, we show that MtMTP2 is a Zn efflux protein located in an intracellular compartment, with an important role in nodule development and bacteroid differentiation.

## Materials and Methods

### Biological Materials and Growth Conditions

*Medicago truncatula* R108 ecotype, the *Tnt1*-insertion mutants *mtp2-1* (NF11171) and *mtp2-2* (NF18305) were used in this study. Seeds were scarified in concentrated H_2_SO_4_ for 7 min and washed in dH_2_O. Later, seeds were surface sterilized using 50% bleach for 90 s, washed in sterile dH_2_O, and left overnight in sterile water to facilitate imbibition. After 48 h at 4°C, seeds were germinated in water-agar plates at 22°C for 48 h. Seedlings were then transplanted to sterilized perlite pots and inoculated with *Sinorhizobium meliloti* 2011, *S. meliloti* 2011 transformed with the GFP expressing pHC60 vector (Cheng and Walker, 1998), or *S. meliloti* 1021 expressing pCMB13 DsRED (Gage, 2002), as indicated. Plants were cultivated in a greenhouse in 16 h of light and 22°C conditions, and watered every 2 days with Jenner’s solution or water, alternatively (Brito et al., 1994). This nutrient solution contained 5 mM CaSO_4_, 1 mM KCl, 1 mM K_2_HPO_4_, 1 mM MgSO_4_, 11.5 μM H_3_BO_3_, 7.3 μM Fe-citrate, 3.6 μM MnSO_4_, 0.38 μM ZnSO_4_, 0.16 mM CuSO_4_, and 4 nM (NH_4_)_6_Mo_2_O_24_. Nodules were collected 28 dpi. Non-nodulated plants were watered every 2 weeks with solutions supplemented with 20 mM NH_4_NO_3_. For hairy-root transformations, *M. truncatula* seedlings were transformed with *Agrobacterium rhizogenes* ARqua1 carrying the appropriate binary vector as described (Boisson-Dernier et al., 2001).

Complementation assays were performed using the yeast (*Saccharomyces cerevisiae*) strains DY1457 (MATa *ade6 can1 his3 leu2 trp1 ura3*), the yeast double mutant *zrc1cot1* (MATa *ade6 can1 his3 leu2 trp1 ura3 zrc1::his3 cot1::ura3*) (MacDiarmid et al., 2000), DY150 (MATa *ade2-1 his3-11 leu2-3,112 trp1-1 ura3-52 can1-100*(*oc*)), the mutant *ccc1* (MATa *ade2-1 his3-11 leu2-3,112 trp1-1 ura3-52 can1-100*(*oc*) *ccc1::his3*; Li et al., 2001), BY4741 (*his3 leu2 met1 ura3*), and the strain *Δsmf1* (*his3 leu2 met1 ura3 Δsmf1*; ThermoFisher). All strains were grown in synthetic dextrose (SD) medium (Sherman et al., 1981) supplemented with necessary auxotrophic requirements, with 2% (w/v) glucose as the carbon source, and supplemented with Zn, iron, or manganese, when required.

### Sequence Analysis and Protein Structure Prediction

To identify *M. truncatula* MTP family members, BLASTN and BLASTX searches were carried out in the *M. truncatula* Genome Project site^1^ and include 13 members: MtMTP1, *Medtr8g024240*; MtMTP2, *Medtr4g064893*; MtMTP3, *Medtr2g036390*; MtMTP4, *Medtr3g080090*; MtMTP5, *Medtr7g093290*; MtMTP6, *Medtr1g088870*; MtMTP7, *Medtr4g008150*; MtMTP8, *Medtr3g062610*; MtMTP9, *Medtr2g064405*; MtMTP10, *Medtr8g046550*; MtMTP11, *Medtr7g022890*; MtMTP12, *Medtr6g463330*, MtMTP13, *Medtr5g075680*. Sequences from model MTP genes were obtained from the Transporter Classification Database^2^ (Saier et al., 2014), NCBI^3^ and Phytozome^4^, and include *A. thaliana MTPs* (AtMTP1, *At2g46800*; AtMTP2, *At3g61940*, AtMTP3, *At3g58810*; AtMTP4, *At2g29410*; AtMTP5, *At3g12100*; AtMTP6, *At2g4783*; AtMTP7, *At1g51610*; AtMTP8, *At3g58060*; AtMTP9, *At1g79520*; AtMTP10, *At1g16310*; AtMTP11, *At2g39450*; AtMTP12, *At2g04620*), *Oryza sativa* MTPs (OsMTP1, *LOC_Os05g03780*; OsMTP8, *LOC_Os05g03780*) *Cucumis sativus* MTPs (CsMTP1, *Cucsa.362220*; CsMTP4, *Cucsa.146570*; CsMTP8, *Medtr3g062610*; CsMTP9, *Cucsa.118550*), *Anemone halleri* MTPs (AhMTP1*-A, FN428855*; AhMTP1*-B, Fn386317*; AhMTP1*-C, Fn386316*; AhMTP1*-D, Fn386315*), *Hordeum vulgare* MTPs (HvMTP1, *HORVU1Hr1G015500*; HvMTP8*.1, HORVU4Hr1G065110.1*), and *Populus trichocarpa* MTPs (PtdMTP1, *Potri.014G106200*; PtMTP11, *POPTR_0010s21810*). All these protein sequences were processed with MEGA7^5^. First, protein sequences were aligned using the Clustal Omega algorithm^6^ (Sievers et al., 2011), and the alignment was visually examined to exclude alignment artifacts. Then, phylogenetic reconstruction was performed using the Neighbor-joining method, the Jones–Taylor–Thornton (JTT) substitution model, and assuming uniform rates (Saitou and Nei, 1987; Jones et al., 1992). Deletion sites were excluded from the alignment following the partial deletion method (95% site coverage cutoff). Unrooted tree visualization was carried out using FigTree^7^.

MtMTP2 protein sequence from R108 was obtained from the Medicago Hapmap website^8^. The automated protein homology-modeling server SWISS-model^9^ (Biasini et al., 2014) was used to predict the MtMTP2 protein structure based on the template 3h90 from the *Escherichia coli* Zn transporter YiiP (Lu et al., 2009). Protein structure was visualized using PyMOL (Schörindeger LLC, United States).

### RNA Extraction and RT-qPCR

RNA was isolated from leaves, roots, or nodules from three-pooled plants (from independent experiments each) following the protocol previously described by Abreu et al. (2017). Briefly, RNA was extracted using Tri-Reagent^®^ (Life Technologies, Carlsbad, CA) followed by a DNase treatment and later cleaned with RNeasy Minikit (Qiagen, Valencia, CA). Denaturing agarose gel was used to verify RNA quality. One microgram of DNA-free RNA was employed to generate cDNA by using PrimeScript RT Reagent Kit (Takara). Gene expression was determined by quantitative Real time RT-PCR (9700, Applied Biosystems, Carlsbad, CA, United States) using primers listed in Supplementary Table S1. The *M. truncatula ubiquitin carboxyl-terminal hydrolase* gene was used to normalize the results. Real-time cycler conditions have been previously described (González-Guerrero et al., 2010). The threshold cycle (Ct) was determined in triplicate. The relative levels of transcription were calculated using the 2^-ΔΔCt^ method (Livak and Schmittgen, 2001). As control, a non-RT sample was used to detect any possible DNA contamination.

### Yeast Complementation Assays

Yeast complementation was performed by cloning the *MtMTP2* cDNA between the XbaI and BamHI sites of the yeast expression vector pAMBV or pDR196. Cloning in pAMBV was carried out by homologous recombination of *MtMT2* cDNA using primers 5 MtMTP2 XbaI pMBV and 3 MtMTP2 BamHI pAMBV (Supplementary Table S1). Cloning in pDR196 was carried out by restriction digestion and T4 ligation of the DNA fragment resulting from the XbaI and BamHI digestion of the amplicon resulting from amplifying by PCR *MtMTP2* cDNA with primers 5MtMTP2-XbaI and 3MtMTP2-BamHI (Supplementary Table S1). Yeast transformations were performed using a lithium acetate-based method (Schiestl and Gietz, 1989). Cells transformed with *pAMBV* or *pAMBV::MtMTP2* (in Zn phenotypic assays) or pDR196 or pDR196 (in the case of iron or manganese phenotypic assays) were selected in SD medium by leucine or uracil autotrophy, respectively. For phenotypic tests, DY1457 and *zrc1cot1* transformants were plated in SD with or without supplementation of 500 μM ZnSO_4_, DY150, and *ccc1* transformants were plated in SD with or without supplementation with 4 mM FeSO_4_ and BY4741 and *Δsmf1* transformants were plated in SD with or without supplementation with 10 mM MnCl_2_.

### GUS Staining

A transcriptional fusion was constructed by amplifying 889 bases upstream of *MtMTP2* start codon using primers indicated on Supplementary Table S1, cloned in pDONR207 (Invitrogen), and transferred to pGWB3 (Nakagawa et al., 2007) using Gateway technology^®^ (Invitrogen). This led to the fusion of the promoter region of *MtMTP2* with the β*-glucoronidase* (*gus*) gene in pGWB3. pGWB3::*MtMTP2* was transformed in *A. rhizogenes* ARqua1 and used to obtain *M. truncatula* composite root plants as indicated (Boisson-Dernier et al., 2001). GUS activity was determined in 28 dpi plants as described (Vernoud et al., 1999). The process was carried out from biological material originated from three independent assays carried out at different times of the year to select a representative image.

### Immunolocalization of MtMTP2-HA

By using Gateway Technology^®^ (Invitrogen), a DNA fragment of the full length *MtMTP2* genomic region and the 1,961 bases upstream of its start codon, was cloned into the plasmid pGWB13 (Nakagawa et al., 2007). Hairy-root transformation was performed as previously described (Boisson-Dernier et al., 2001). For confocal microscopy, transformed plants were inoculated with *S. meliloti* 2011 containing the pHC60 plasmid that constitutively expresses GFP (Cheng and Walker, 1998) or DsRED (Gage, 2002). Roots and nodules collected from 28 dpi plants were fixed by overnight incubation in 4% paraformaldehyde, 2.5% sucrose in phosphate buffer saline (PBS) at 4°C. After washing in PBS, nodules were cut in 100 μm sections with a Vibratome 1000 plus (Vibratome, St. Louis, MO, United States). Sections were dehydrated in a methanol series (30, 50, 70, 100% in PBS) for 5 min and then rehydrated. Cell walls were treated with 4% cellulase in PBS for 1 h at room temperature and with 0.1% Tween 20 in PBS for an additional 15 min. Sections were blocked with 5% bovine serum albumin (BSA) in PBS before their incubation with an anti-HA mouse monoclonal antibody (Sigma, St. Louis, MO) for 2 h at room temperature. After washing, an Alexa 594-conjugated anti-mouse rabbit monoclonal antibody (Sigma) was added to the sections for 1 h at room temperature. DNA was stained with DAPI after washing. Images were acquired with a confocal laser-scanning microscope (Leica SP8, Wetzlar, Germany). The process was carried out from biological material originated from three independent assays carried out at different times of the year to select a representative image.

Immunolocalization of MtMTP2-HA in an electron-microscope was carried out with *M. truncatula* plants transformed with *A. rhizogenes* ARqua1 pGWB13 carrying *MtMTP2* full gene and the 1,961 bases upstream the start codon. Transformed plants were inoculated with *S. meliloti* 2011 and 28 dpi nodules were collected and fixed in 1% formaldehyde and 0.5% glutaraldehyde in 50 mM potassium phosphate (pH 7.4) for 2 h. After that, the fixation solution was renewed for 1.5 h. Samples were washed in 50 mM potassium phosphate (pH 7.4) 3 × 30 min and 3 × 10 min. Nodules were dehydrated by incubation with ethanol dilution series of 30, 50, 70, 90 (10 min each), 96 (30 min), and 100% (1 h). Nodules were included in a series of ethanol and LR–white resin (London Resin Company Ltd., United Kingdom) dilutions: 1:3 (3 h), 1:1 (overnight), and 3:1 (3 h). Samples were included in resin during 48 h. All the process was performed at 4°C. Nodules were placed in gelatine capsules and filled with resin and polymerized at 60°C for 24 h. One-micron thin sections were prepared at *Centro Nacional de Microscopía Electrónica* (Madrid, Spain) with a Reichert Ultracut S-ultramicrotome fitted with a diamond knife. Thin sections were blocked in 2% bovine serum albumin in PBS for 30 min. Anti-HA rabbit monoclonal antibody (Sigma) was used as primary antibody, a 1:20 dilution in PBS. Samples were washed 10 times in PBS for 2 min. Anti-rabbit goat conjugated to a 15-nm gold particle (BBI solutions) was used as secondary antibody diluted 1:150 in PBS. Incubation was performed for 1 h followed by 10 washes in PBS for 2 min and 15 times in water for 2 min. Sections were stained with 2% uranyl acetate and imaged in a JEM 1400 electron microscope at 80 kV.

### Acetylene Reduction Assay

Nitrogenase activity was measured by the acetylene reduction assay (Hardy et al., 1968). Nitrogen fixation was assayed in 28 dpi wild-type and mutant plants in 30 ml tubes fitted with rubber stoppers. Each tube contained roots from five independently transformed plants. Three milliliters of air inside were replaced with 3 ml of acetylene. Tubes were incubated at room temperature for 30 min. Gas samples (0.5 ml) were analyzed in a Shimadzu GC-8A gas chromatograph fitted with a Porapak N column. The amount of ethylene produced was determined by measuring the height of the ethylene peak relative to background. Each point consists of three tubes each with five pooled plants measured in triplicate.

### Metal Content Determination

Inductively coupled plasma mass spectrometry (ICP-MS) was carried out at the Metal Analysis Unit of the Scientific and Technology Centre, Universidad de Barcelona (Barcelona, Spain). These samples were digested with HNO_3_, H_2_O_2_, HF in a Teflon reactor at 90°C. The sample was diluted with deionized water. The final volume of the solution was calculated by weight difference with the original sample, and with the measured density of the solution, obtained from weighting a small aliquot of known volume. Samples were digested with three blanks in parallel. Metal determination was carried out in Agilent 7500ce instrument under standard conditions (RF power 1550 W, Nebulizer Burgener AriMist HP, Nebulizer Ar flow 0.75 l/min, sample pump 0.1 rps, QP resolution 0.7 amu at 10% height (^7^Li, ^89^Y, ^205^Tl), integration time 0.9 s, reading replicates 3, calibration linear through zero, internal standard online addition ^103^Rh, gas cell mode He collision). Calibration was carried out with five measurements using commercial certified solutions analyzed and compared with reference NIST solutions.

### Confocal Imaging of Bacteroids and Colony-Forming-Units Assays

Confocal microscopy images of bacteroids were obtained from 28 dpi nodules. Nodules were ground with a micropestle in TY medium to release the bacteroids. The nodule homogenate was filtered using CellTrics^®^ 30 μm columns and stained using propidium iodide (50 μg/ml) to visualize the bacteroids by using confocal microscopy at a 535 nm Ex/617 nm Em. Colony-forming-units (CFU) were obtained from fresh nodules harvested at 28 dpi. Nodules were weighed and surface-sterilized in 70% ethanol for 10 min followed by five washes with distillated water. The tissue was later ground with a micropestle in 200 μl TY medium. Serial dilutions of the homogenate were plated on TY solid media. Plates were incubated 48 h at 30°C, and the number of colonies was recorded (Montiel et al., 2016).

### Statistical Tests

Results are presented as mean value ± standard deviation. Multiple comparisons were performed by one-way analysis of variance (ANOVA) followed by Tukey HSD *post hoc* at a probability level of 5% (*P* < *0.05*). Pairwise comparisons were done by using Student’s *t*-test at a probability level of 5% (*P* < *0.05*). The JMP^®^ (ver.11.0) statistical package (SAS Institute) was used for statistical analyses.

## Results

### MtMTP2 Is Up-Regulated During Nodule Development

**FIGURE 1:**
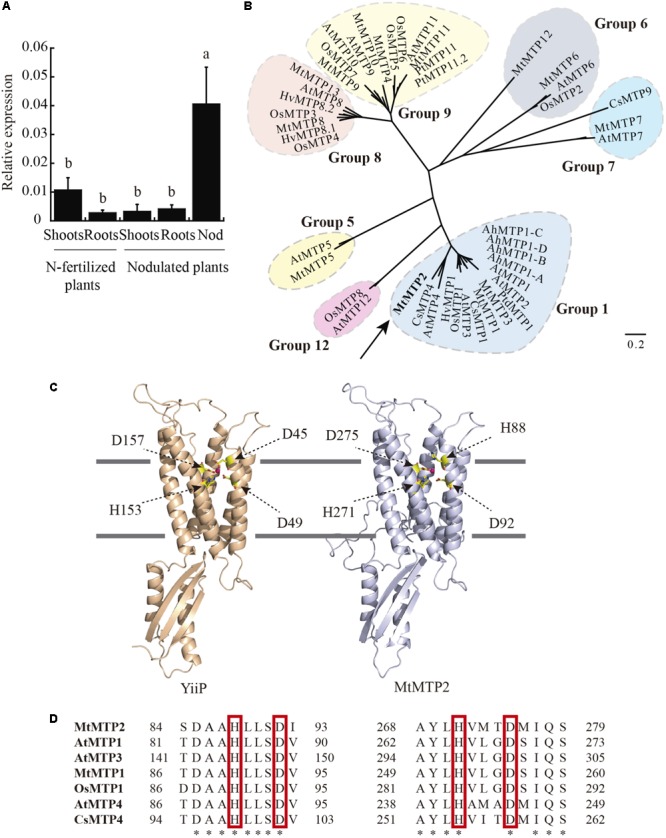
*Medicago truncatula* metal tolerance protein 2 (MtMTP2), a member of the MTP protein family, is highly expressed in the nodule. **(A)** *MtMTP2* expression in nitrogen-fertilized and 28 dpi nodulated *M. truncatula* plants relative to the internal standard gene *Ubiquitin carboxyl-terminal hydrolase*. Values with different letters are significantly different (Tukey’s HSD *p* < 0.05; *n* = 4). **(B)** Unrooted phylogenetic tree of the plant metal tolerance protein family. **(C)** Tertiary structure model of MtMTP2 and its template YiiP (3h90). Positions of metal coordinating amino acids and Zn(II) elements are indicated. **(D)** Alignment of the conserved amino acids in MTP members. The amino acid sequences were aligned using the ClustalW method.

Out of the thirteen MTP genes in the *M. truncatula* genome, MtMTP2 was the one with the highest expression levels in nodules, as reported in the Medicago Gene Expression Atlas (Benedito et al., 2008) and in the Symbimics database (Roux et al., 2014; Supplementary Figure S1). *MtMTP2* expression analysis was performed to identify organs in which the gene/protein operates. Relatively high levels of *MtMTP2* transcripts were found in nodules compared to shoots and roots of inoculated plants (**Figure 1**). Shoots of nitrogen-fertilized, non-nodulated plants exhibited higher transcript levels than those of nodulated plants, although levels were still much lower than in nodules (**Figure 1**).

MTP proteins fall into seven groups, based on sequence similarity, which roughly correspond to putative metal substrates and subcellular localizations (Ricachenevsky et al., 2013). To gain insight into the possible metal substrates of MtMTP2, phylogenetic analysis was performed on 13 *M. truncatula* MTPs and homologous proteins in *Arabidopsis thaliana, Oryza sativa, Crocus sativus, Arabidopsis halleri, Populus trichocarpa, and Hordeum vulgare.* MtMTP2 showed strong similarity to CsMTP4 (54% similarity) described as a protein involved in Zn homeostasis in cucumber (Migocka et al., 2015) and AtMTP4 (48% similarity), a predicted Zn transporter of *A. thaliana* (Waters and Grusak, 2008; **Figure 1B**). To explore further Zn as a candidate substrate, a predicted tertiary structure of MtMTP2 was obtained by homology modeling based on the known crystal structure of the *E. coli* Zn transporter YiiP (Lu et al., 2009). The generated model revealed a Zn-binding domain made of residues H88, D92, H271, and D275 that corresponded to the YiiP site Z1 (D45, D49, H153, and D157; **Figure 1C**). The substitution of YiiP D45 residue by a histidine, H88, observed in MtMTP2 is also conserved in other plant MTP proteins (**Figure 1D**).

### MtMTP2 Complements a Zinc Detoxification-Deficient Yeast Mutant

**FIGURE 2:**
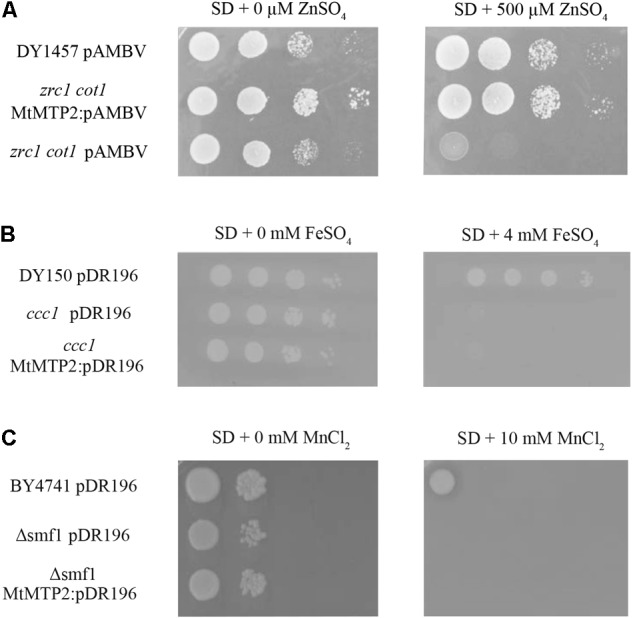
MtMTP2 yeast complementation assays. **(A)** Yeast strain DY1457 was transformed with the pYPGE15 empty vector, while the double mutant *zrt1*/*cot1* was transformed with the empty pYPGE15 or with pYPEG15 containing *MtMTP2* coding DNA sequence. Serial dilutions (10×) of each transformant were grown for 3 days at 28°C on SD media with all the required amino acids and 500 μM ZnSO_4_. Positive control was obtained without supplementing the media with ZnSO_4_. **(B)** Yeast strain DY150 was transformed with the pDR196 empty vector, while *ccc1* mutant was transformed either with the empty pDR196 or with pDR196 containing *MtMTP2* coding DNA sequence. Serial dilutions (10×) of each transformant were grown for 3 days at 28°C on SD media with all the required amino acids and 4 mM FeSO_4_. Positive control was obtained without supplementing the media with FeSO_4_. **(C)** Yeast strain BY4741 was transformed with the pDR196 empty vector, while a deletion strain in *smf1* was transformed either with empty pDR196 or with pDR196 containing *MtMTP2* coding DNA sequence. Serial dilutions (10×) of each transformant were grown for 3 days at 28°C on SD media with all the required amino acids and 10 μM MnCl_2_. Positive control was obtained without MnCl_2_ supplementation.

In *S. cerevisiae*, ZRC1 and COT1 are tonoplast transporters responsible for the storage of Zn in vacuoles (MacDiarmid et al., 2000). Yeast *zrc1/cot1* double mutants are hyper-sensitive to Zn in the growth medium. Genetic complementation assays using a yeast *zrc1/cot1* double mutant showed that expression of *MtMTP2* enabled the mutant strain to grow on otherwise toxic levels of Zn (500 μM ZnSO_4_), consistent with a role of MtMTP2 in Zn efflux out of the cytosol (**Figure 2A**). In contrast, complementation assays using a yeast *ccc1* mutant affected in the transport of iron into the vacuole (Li et al., 2001), and an *smf1* mutant strain, which is unable to store manganese in the vacuole (Portnoy et al., 2000), showed no recovery of growth on media supplemented with 4 mM FeSO_4_ or 10 mM MnCl_2_, respectively (**Figures 2B,C**), indicating that MtMTP2 does not transport Fe or Mn in yeast.

### MtMTP2 Is Located in an Endomembrane Compartment in Cells of Nodule Zones II to III

**FIGURE 3:**
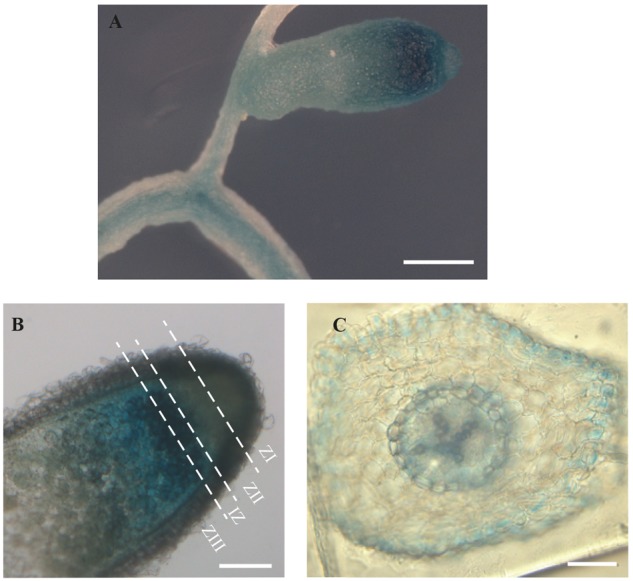
Organ and tissue expression localization of *MtMTP2*. **(A)** GUS expression assay in roots and nodules of plants transformed with pGWB3 containing the *pMtMTP2::GUS* construct. Bar: 1 μm. **(B)** GUS expression assay in a longitudinal section of a nodule transformed with pGWB3 containing *pMtMTP2::GUS* construct. Bar: 100 mm. **(C)** Cross section of a *M. truncatula* root transformed with the plasmid pGWB3 containing the *pMtMTP2::GUS* construct. Bar: 50 μm.

To determine *MtMTP2* expression distribution in nodules and roots, a segment of 889 bp upstream of the *MtMTP2* start codon was fused to the *gus* reporter gene and subsequently expressed in roots of *M. truncatula* inoculated with rhizobia. *MtMTP2* promoter activity was detected in roots and nodules (**Figure 3A**) and was most active in the segment from late Zone II to early Zone III, while lower GUS signal was detected at the meristematic zone and late zone III (**Figure 3B**). *In silico* analysis of *MtMTP2* expression in nodules using data obtained from the Symbimics database^10^ (Roux et al., 2014) was consistent with GUS assays showing an increased expression pattern in the late differentiation zone (proximal Zone II) and in Zone III (Supplementary Figure S2). In roots, *MtMTP2*-regulated GUS activity was faintly detected at the epidermis, pericycle, and vascular tissue (**Figure 3C**).

**FIGURE 4:**
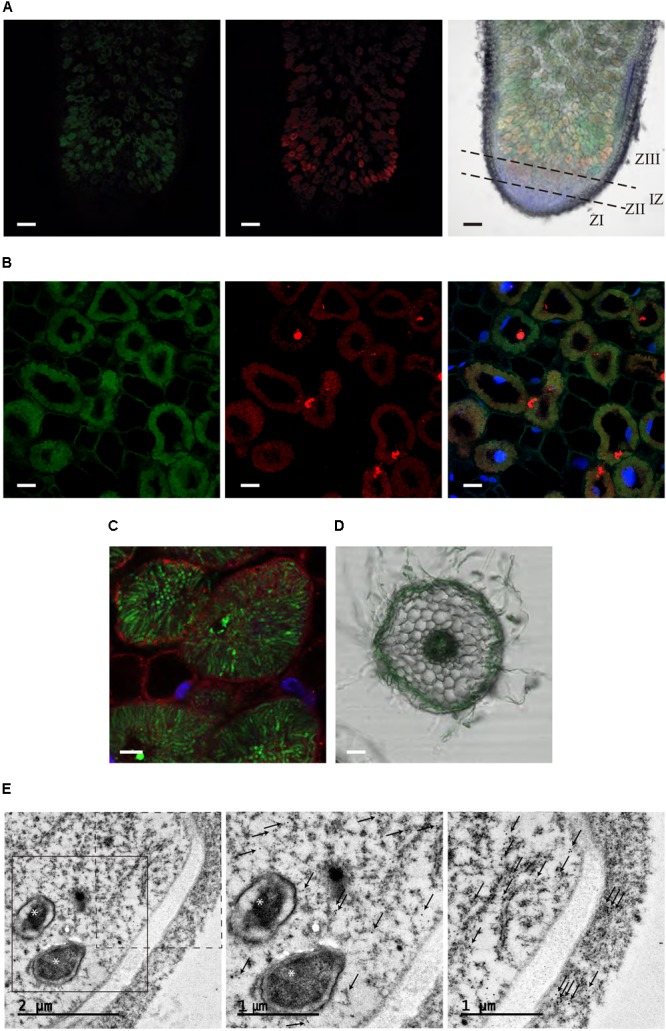
Subcellular localization of *MtMTP2* in *Medicago truncatula*. **(A)** Cross section of a 28-dpi *M. truncatula* nodule expressing *pMtMTP2::MtMTP2-GFP* (green) **(A)** inoculated with a *Sinorhizobium meliloti* 1021 strain constitutively expressing DsRed (red). DNA was stained using 4′-6-diamino-phenylindole (DAPI) (blue). Left panel, GFP channel; central panel, DsRed channel; right panel, overlay of the previous two panels with DAPI channel and transillumination image. Bars: 100 μm. **(B)** Closer view of infection zone of a 28-dpi *M. truncatula* nodule expressing *pMtMTP2::MtMTP2-GFP* (green) inoculated with a *Sinorhizobium meliloti* 1021 strain constitutively expressing DsRed (red). DNA was stained using 4′-6-diamino-phenylindole (DAPI) (blue). Left panel, GFP channel; central panel, DsRed channel; right panel, overlay of the previous two panels with DAPI channel image. Bars: 25 μm. **(C)** Detail of an infected area of *M. truncatula* nodules transformed with *pMtMTP2::MtMTP2-HA*. Sections were immunostained with the antibody Alexa 594 (red). Transformed plants were inoculated with *S. meliloti* 2011 pHC60 strain constitutively expressing GFP (green). Bars: 10 μm. **(D)** Cross section of a 28-dpi *M. truncatula* root transiently expressing *pMtMTP2::MtMTP2-GFP* (green) overlaid with transillumination image. Bar: 50 μm. **(E)** Transmission electron microscopy (TEM) image of an infected cell of a 28 dpi *M. truncatula* nodule expressing the *pMtMTP2::MtMTP2-HA* construct, inoculated with *S. meliloti* 2011. Gold particles are indicated by arrows; bacteroids are indicated by asterisks. Left panel, general overview of an infected (left) and un-infected (right) cells; central panel, closer view of the region boxed with continuous line from the previous panel; right panel, closer view of the region boxed with discontinuous line in the left panel.

Subcellular localization of *MtMTP2* was performed by fusing the DNA segment from 1,961 bp upstream of the *MtMTP2* start codon to the last codon before the stop codon to either GFP or to 3 hemagglutinin (HA) epitopes, resulting in *pMtMTP2::MtMTP2-GFP* and *pMtMTP2:: MtMTP2-HA*, respectively. Plants were transformed with these constructs and inoculated with *S. meliloti* strains constitutively expressing DsRED or GFP, followed by DAPI staining (**Figure 4**). Subcellular localization using both constructs was consistent with the results obtained with the GUS reporter assays. *pMtMTP2::MtMTP2-GFP* was localized intracellularly from the infection and differentiation zones and into the fixation zone in 28 dpi nodules, while no signal was found at the meristematic zone (**Figure 4A**). Localization using the *pMtMTP2::MtMTP2-HA* construct by immunostaining with Alexa594-conjugated antibody (Supplementary Figure S3), showed an identical pattern of distribution to that observed with *pMtMTP2::MtMTP2-GFP*. High magnification imaging of nodules 28 dpi allowed the visualization of *MtMTP2* intracellularly in infected cells (**Figures 4B,C**). No autofluorescence signal was detected under the experimental conditions used, as shown when primary anti-HA antibody for MtMTP2-HA detection was removed (Supplementary Figure S4), or when no MtMTP2-GFP protein was present (Supplementary Figure S5). In roots, MtMTP2-HA was detected in the epidermis and around the vasculature (**Figure 4D**), consistent with the promoter-GUS assays.

To further clarify the subcellular localization of MtMTP2, immunolocalization of MtMTP2-HA with a gold-conjugated antibody and transmission electron microscopy was used (**Figure 4E**). Gold particles were found concentrated in electron dense structures corresponding to intracellular compartments resembling endoplasmic reticulum (or associated domains) of infected cells and non-infected cells. No gold particles were detected associated with symbiosomes. When no primary antibody was used, no gold particles were found to be concentrated in any cell sections (Supplementary Figure S6).

### MtMTP2 Mutants Exhibit Abnormal Accumulation of Zinc in Nodules and Impaired Nitrogen Fixation and Growth

**FIGURE 5:**
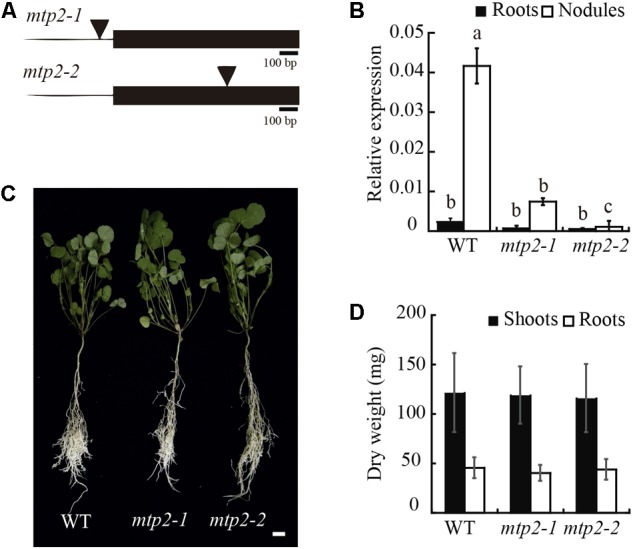
*MtMTP2* mutants grown under non-symbiotic conditions did not show an altered phenotype. **(A)** Position of the transposable element of tobacco (*Nicotiana tabacum*) cell type 1 (*Tnt1*) in the *MtMTP2* genomic region in *mtp2-1* and *mtp2-2* mutants. **(B)** *MtMTP2* expression in roots (black) and nodules (white) of WT, *mtp2-1*, and *mtp2-2* plants relative to the internal gene *Ubiquitin carboxyl-terminal hydrolase 1*. Data are the mean ± SD of two independent experiments with four pooled plants. **(C)** From the left to the right, representative plants of *M. truncatula* WT, *mtp2-1*, and *mtp2-2* N-fertilized plants. Bars: 1 cm. **(D)** Dry weight of shoots (black) and roots (white). Data represent the mean ± SD of three experiments pooling, at least, ten independent plants (*n* = 10).

To determine the physiological role of MtMTP2, two homozygous mutant lines of *MtMTP2, mtpt2-1* (NF11171), and *mtp2-2* (NF18305) were evaluated. Mutant line *mtpt2-1* harbors the *tnt1* insertion at the promoter region (-69 upstream of the start codon) while *mtp2-2* contains a *tnt1* insertion at the unique exon of the gene (+623 downstream the start codon; **Figure 5A**). These insertions resulted in a reduced level of *MtMTP2* expression in nodules, 80% reduction in the case of *mtpt2-1* and more than 99% in *mtpt2-2* (**Figure 5B**). Accordingly, they were designated as a knock-down and a knock-out *MtMTP2* mutants, respectively. Plant phenotypes were analyzed when nitrogen was provided in the nutrient solution as ammonium nitrate. Under these non-symbiotic conditions, no changes in the plant phenotype were detected in the *mtp2* mutants compared to wild-type plants (**Figure 5C**). Plant biomass (determined by the dry weight of shoots and roots) did not show significant differences among the genotypes analyzed (**Figure 5D**). Similarly, no significant differences between wild type and mutant lines were observed when either no Zn or excess Zn (100×) were added to the nutrient solution (Supplementary Figure S7).

**FIGURE 6:**
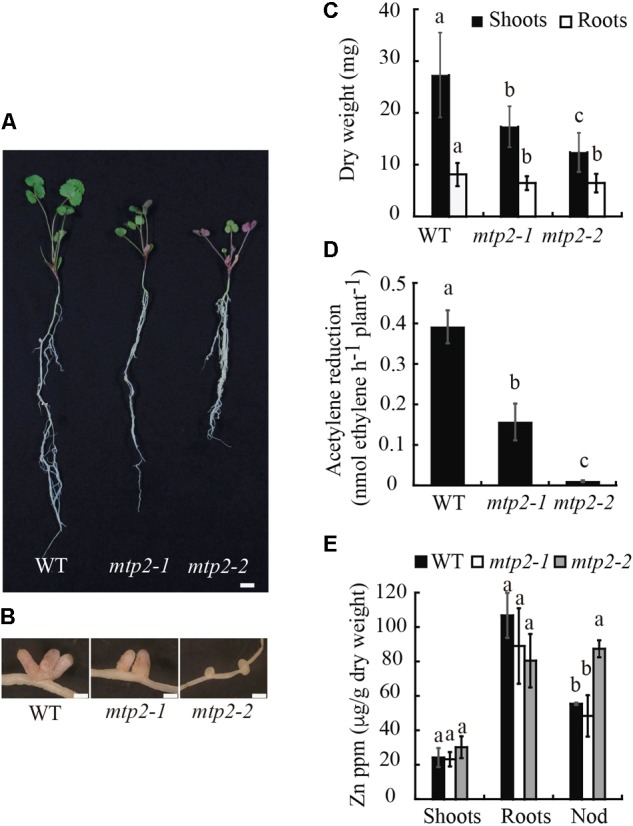
*Medicago truncatula MTP2* mutation impairs nitrogen fixation. **(A)** Representative WT, *mtp2-1*, and *mtp2-2* plants. Bars: 1 cm. **(B)** Representative nodules of WT, *mtp2-1*, and *mtp2-2* plants. Bars: 500 μm. **(C)** Dry weight of shoots (black) and roots (white). Data are the mean ± SD of, at least, 10 plants. Comparisons have been made among the shoots and among the roots. **(D)** Nitrogenase activity of 28-dpi nodules. Acetylene reduction was measured in duplicate from three sets of four pooled plants. Data are the mean ± SD. **(E)** Zn content in shoots, roots and nodules of WT (black), *mtp2-1* (white), and *mtp2-2* (gray). Data are the mean ± SD of three sets of at least ten pooled plants. Values with different letters are significantly different (Tukey’s HSD, *p < 0.05*).

In contrast, under symbiotic conditions without mineral-N, mutant plants exhibited reduced growth and an altered nodule development (**Figure 6**). Nodules of the *mtp2-2* mutant were small, round, and white, in contrast to the long, cylindrical, pink nodules of the wild-type (**Figures 6A,B**). A time-course analysis of nodule growth was performed, which showed a progressive delay in nodule growth, but no delay in the start of nodulation of the mutants (Supplementary Figure S8). Consistent with the plant phenotypes observed, plant biomass (determined as dry weight) was reduced in both mutants (**Figure 6C**). Shoot biomass suffered a more drastic decrease linked to the mutation than did root biomass (a 36% reduction in the case of *mtpt2-1* and 55% for *mtpt2-2* shoots). However, root biomass was still significantly diminished in both mutants, and to a similar extent (20%). In order to determine if the phenotypic alterations observed were a consequence of a decline in nitrogenase activity, acetylene reduction assays were performed (Hardy et al., 1968). The knock-down mutant, *mtpt2-1* exhibited a reduction of 60% in nitrogenase activity while the knock-out mutant, *mtpt2-2* barely showed detectable enzyme activity (**Figure 6D**). Similar activity profile was observed when data were normalized to nodule number per root (Supplementary Figure S9). Zn content in shoots, roots, and nodules were determined in order to evaluate changes in the putative metal substrate of MtMTP2. While no significant changes in Zn content were detected in roots, and a slight increase was detected in the shoots of *mtpt2-2*, nodules of knock-out plants showed a substantial accumulation of Zn (∼40% increase; **Figure 6E**). This phenotype was not the result of additional insertions in the *tnt1* lines. Both mutant lines only share insertions in *MtMTP2.* Moreover, segregants containing the two wild-type copies of *MtMTP2* did not show any significant differences with wild-type plants (Supplementary Figure S10). A Zn gradient including suboptimal (0 μM added ZnSO_4_), control (0.38 μM ZnSO_4_) and supra-optimal Zn conditions (38 μM ZnSO_4_) was applied in an attempt to complement the *mtp2* mutants’ defective symbiotic phenotype (Supplementary Figure S11). None of the Zn conditions tested enabled recovery in growth (including biomass; Supplementary Figure S11A,B) nor in nitrogenase activity (Supplementary Figure S11C).

### Absence of MTP2 Alters Nodule and Bacteroid Development

**FIGURE 7:**
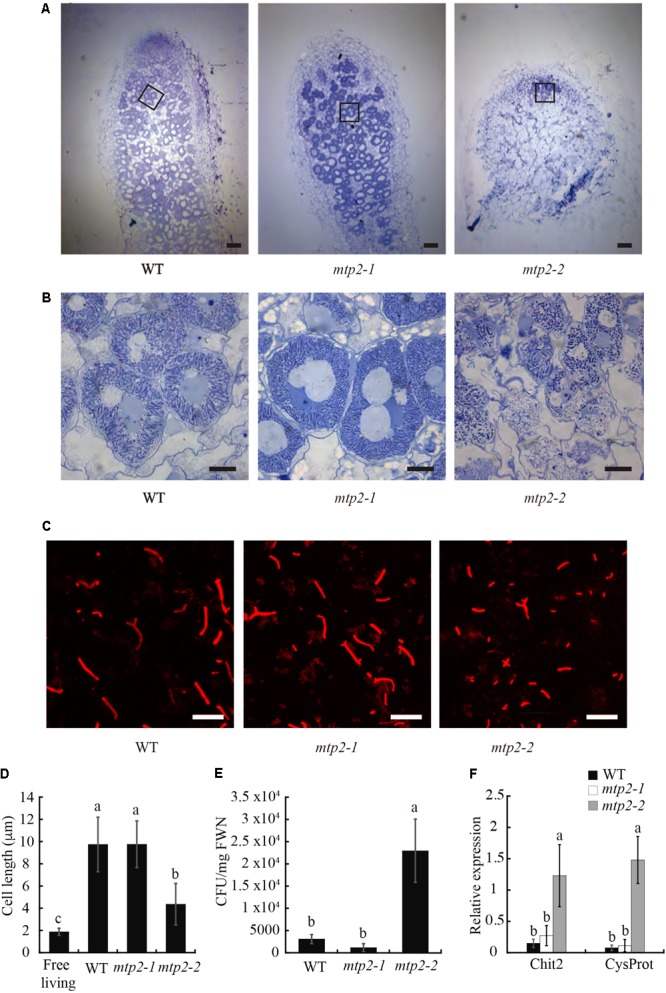
Nodule and bacteroid development in *MtMTP2* mutants. **(A)** Longitudinal sections of *M. truncatula* WT, *mtp2-1*, and *mtp2-2* nodules stained with toluidine blue and visualized by light microscopy. Bars: 100 μm. **(B)** Detail of infected nodule cells of *M. truncatula* WT, *mtp2-1*, and *mtp2-2* nodules stained with toluidine blue and visualized by light microscopy. Bars: 10 μm. **(C)** Confocal image of *S. meliloti* stained with propidium iodide from 28-dpi *M. truncatula* WT, *mtp2-1*, and *mtp2-2* nodules. **(D)** Cell length of *S. meliloti* bacteroids isolated from WT, *mtp2-1*, and *mtp2-2* nodules. Data are the mean ± SD of at least 100 cells. **(E)** *S. meliloti* CFU per mg fresh weight of 28-dpi nodules from WT, *mtp2-1*, and *mtp2-2* plants. Data are the mean of three independent experiments ± SE. Values with different letters are significantly different (Tukey’s HSD, *p* < 0.05). **(F)** Expression of the senescence marker genes Chit2 (chitinase *Medtr5g022560*) and CysProt (cysteine protease *Medtr6g079630*) in 28 dpi *M. truncatula* nodules of WT, *mtp2-1*, and *mtp2-2* plants relative to the internal standard gene *Ubiquitin carboxyl-terminal hydrolase*. Data are the mean of three independent experiments ± SE. Values with different letters are significantly different (Tukey’s HSD, *p* < 0.05).

The smaller size of *mtp2* mutant nodules indicated altered nodule development. To explore this further, nodules were sectioned, stained with toluidine blue, and observed by light microscopy. Nodules of *mtpt2-2* a reduced infection zone and the disappearance of the fixation zone compared to WT nodules (**Figures 7A,B**).

The lack of a fixation zone in *mtp2-2* suggested that rhizobia may not have differentiated fully into bacteroids in these nodules. To test this idea, bacteroids were isolated from WT and *mtp2* mutant nodules and characterized (**Figures 7C,D**). Bacteroids from *mtp2-2* nodules were shorter in length than those of wild-type nodules, longer than free-living rhizobia (**Figure 7D**) and formed more colonies on solid growth medium (**Figure 7E**). These results are consistent with lack of full differentiation of rhizobia into nitrogen-fixing bacteroids within *mtp2-2* mutant nodules, or with early senescence that would hamper further nodule development. To test the later, gene expression of a chitinase (*Medtr5g022560*) and cystine protease (*Medtr6g079630*), two genes induced in senescence (Xi et al., 2013), was determined in 28 dpi nodules from wild-type and *mtp2-1* and *mtp2-2* plants (**Figure 7F**). The result showed an induction of senescent genes in *mtpt2-2* nodules.

## Discussion

MTP proteins are members of the Cation Diffusion Facilitator (CDF) family and known in plants (Ricachenevsky et al., 2013). These proteins are typically involved in Zn^2+^, Mn^2+^, or Fe^2+^ efflux from the cytosol, either out of the cell or into organelles (Desbrosses-Fonrouge et al., 2005; Eroglu et al., 2015). From a physiological point of view, their functions can be diverse, including metal detoxification (Desbrosses-Fonrouge et al., 2005), metal storage and allocation to sink organs (Eroglu et al., 2017), and metalation of apo-metalloproteins (Ellis et al., 2004). From a structural point of view, they seem to function as homodimers (Wei et al., 2004; Lu and Fu, 2007; Lu et al., 2009), in which each of the monomers is able to independently pump cations. This is made possible by transmembrane metal binding sites that, by being arranged in a specific geometry, confer specificity to the transporter (Argüello et al., 2012).

The genome of *M. truncatula* encodes 13 MTP proteins of which only one, MtMTP1, was previously characterized (Chen et al., 2009). MtMTP1 is a Zn transporter also involved in Zn efflux from the cytosol. It is expressed in roots and shoots. Zn modulates *MtMTP1* transcription: in roots, it is down-regulated, while it is up-regulated in shoots in response to Zn supply. Although there is no published information on its expression in nodules, its overall expression decreases with *S. meliloti* inoculation, and the ˆMedicago Gene Expression Atlas and the Symbimics databases indicate that it is downregulated in nodules. In this manuscript, we characterized MtMTP2 as an additional Zn^2+^-transporting MTP family member that is involved in nodule development. Homology modeling of the structure of MtMTP2 shows that its metal substrate would likely be tetrahedrally coordinated by two histidine and two glutamate residues, since they occupy a similar location to the transmembrane metal binding site of template *E. coli* YiiP (Lu and Fu, 2007; Lu et al., 2009). Three of the four amino acid residues are conserved between both proteins, and the fourth, a change from glutamate to histidine, is consistent with Zn binding, both by its occurrence in many Zn-coordinating sites, and by its being conserved in other plant Zn-transporting MTPs (Ricachenevsky et al., 2013). Further supporting this ability to transport Zn are two related observations: the capability of MtMTP2 to functionally complement the Zn transport defect of the yeast *zrc1/cot1* double mutant (MacDiarmid et al., 2000); as well as the changes in Zn concentration in nodules of the *mtp2-2* mutant line. Although MTP proteins have been shown to be able to transport more than one substrate, MtMTP2 did not complement defects in Fe or Mn transport of specific yeast mutants (Podar et al., 2012; Migocka et al., 2015; Eroglu et al., 2017).

*MtMTP2* is expressed in different plant organs. In roots, it is located in the epidermal and in vascular and endodermal cells; in nodules, in cells in Zones II and III, as indicated by promoter::*gus* fusions, fluorescence of a GFP-labeled MtMTP2, and immunolocalization of a HA-tagged protein. The latter two approaches also provide insight into the subcellular localization of MtMTP2, which is associated with an endomembrane compartment. Given the importance of Zn in symbiotic nitrogen fixation (Ibrikci and Moraghan, 1993; O’Hara, 2001), and the direction of transport of MTPs in general and MtMTP2 in particular (Wei and Fu, 2006), it was tempting to speculate that MtMTP2 might deliver Zn across the symbiosome membrane. However, electron microscopy analyses of the localization of HA-tagged MtMTP2 indicated that this is not the case, since no protein was associated with symbiosomes. Instead, signal was located in electron-dense bodies in the cell cytosol, that are distributed all over the cells. This allows us to discard plastids or mitochondria as putative locations based on their unique morphology, as well as the nucleus (based on its uniqueness), Golgi cisternae (very few in a cell), or late endosomal compartment (with a close distribution to the plasma membrane). Putative localization would be the endoplasmic reticulum. Considering that transport into the vacuole would mean a role in Zn storage, rather than a more active role in the cell functioning, and the severe phenotype observed by *MtMTP2* mutation, it can be speculated that the intracellular compartment would correspond to the endoplasmic reticulum. Previously, other MTP/CDF proteins have been associated to the endoplasmic reticulum, where they would play a role in metallating metalloproteins. Mutation of yeast *Msc2* gene results in the induction of the unfolded protein response, as a consequence of the Zn cofactor not being inserted in the proteins (Ellis et al., 2004, 2005). Similar roles of CDF proteins being involved in metallation of proteins have been attributed to *Schizosaccharomyces pombe* Zhf1 (Choi et al., 2018), or to mammalian ZnT5 and ZnT6 (Suzuki et al., 2005). However, co-localization with ER-specific markers would be needed to conclusively demonstrate MtMTP2 subcellular localization.

In spite of being expressed in many plant organs, MtMTP2 is primarily involved in nodule development. No aberrant phenotype was observed for *mtp2* mutants under non-symbiotic conditions, indicating that either MtMTP2 is not required for key physiological processes in plants in the vegetative stage of growth when watered with NH_4_NO_3_, or that another, yet-to-be-determined protein can serve as a substitute for MTP2. However, when nitrogen is provided by endosymbiotic rhizobia in root nodules, mutating MtMTP2 has a dramatic effect. As was the case with silencing the Zn transporter *MtZIP6* (Abreu et al., 2017), altering Zn homeostasis in the nodule resulted in reduced nodule development and a substantial decrease in nitrogenase activity. However, the effect of mutating *MtMTP2* was more severe because its loss lead to alterations in nodule development, in bacteroid maturation, and nodule senescence. Phenotypic differences between *MtZIP6* silenced plants and the *MtMTP2* knock-out mutant seem to result from the inability to completely silence gene expression, since an activity of just 40% in the knock-down *mtp2-1* line is enough to allow for bacteroid development, a situation similar to what was reported for *MtZIP6* RNAi plants. This result is striking since it indicates the existence of one or several Zn proteins that receive Zn in an endomembrane compartment (likely the endoplasmic reticulum) that have an effect on bacteroid differentiation and/or nodule development, leading to early senescence. Alternatively, it could be argued that MtMTP2 might be protecting the nodule against Zn toxicity by sequestering the metal, as has been proposed for other MTP transporters (Blaudez et al., 2003; Desbrosses-Fonrouge et al., 2005). However, when no Zn was provided in the nutrient solution, no improvement of the mutant phenotype was observed, suggesting that no toxicity effect was at play. Moreover, altered nodule development has also been reported when mutating iron transporter SEN1 in *Lotus japonicus* (Hakoyama et al., 2012). Lowering Zn levels in the nutrient solution did not have any effect on plant growth or nitrogen fixation. This could be due to not being able to diminish enough the Zn levels (traces in perlite) or to already having achieved the bare minimum nitrogenase activity in *mtp2-2* plants. Future work will be directed toward characterizing the nodulation Zn-proteome to identify the Zn-proteins that might be governing nodule development and bacteroid differentiation.

## Author Contributions

JL-M and MS carried out most of the experimental work with assistance from ÁS (yeast complementation), PG-D (nodule development time course and effect of added metals on mtp2-2 phenotype), and JM (bacteroid development). IK and MU obtained the mtp2-1 and mtp2-2 mutants. MR, JI, and MG-G were responsible for experimental design, data analyses, and wrote the manuscript with contributions from all authors.

## Conflict of Interest Statement

The authors declare that the research was conducted in the absence of any commercial or financial relationships that could be construed as a potential conflict of interest.

## Footnotes

1 http://www.jcvi.org/medicago/index.php

2 http://www.tcdb.org/

3 http://www.ncbi.nlm.nih.gov/

4 https://phytozome.jgi.doe.gov/pz/portal.html

5 http://www.megasoftware.net

6 https://www.ebi.ac.uk/Tools/msa/clustalo/

7 http://tree.bio.ed.ac.uk/software/figtree/

8 www.medicagohapmap.org

9 https://swissmodel.expasy.org

10 https://iant.toulouse.inra.fr/symbimics/

## References

[B1] Abreu I. Saez A. Castro-Rodríguez R. Escudero V. Rodríguez-Haas B. Senovilla M. (2017). *Medicago truncatula* Zinc-Iron Permease6 provides zinc to rhizobia-infected nodule cells. *Plant Cell Environ.* 40 2706–2719. 10.1111/pce.13035 28732146

[B2] Alloway B. J. (2008). *Zinc in Soils and Crop Nutrition* 2nd Edn. Brussels: International Zinc Association and International Fertilizer Industry Association.

[B3] Argüello J. M. Raimunda D. González-Guerrero M. (2012). Metal transport across biomembranes: emerging models for a distinct chemistry. *J. Biol. Chem.* 287 13510–13517. 10.1074/jbc.R111.319343 22389499 PMC3340188

[B4] Assunção A. G. L. Herrero E. Lin Y.-F. Huettel B. Talukdar S. Smaczniak C. (2010). *Arabidopsis thaliana* transcription factors bZIP19 and bZIP23 regulate the adaptation to zinc deficiency. *Proc. Natl. Acad. Sci. U.S.A.* 107 10296–10301. 10.1073/pnas.1004788107 20479230 PMC2890486

[B5] Benedito V. A. Torres-Jerez I. Murray J. D. Andriankaja A. Allen S. Kakar K. (2008). A gene expression atlas of the model legume *Medicago truncatula*. *Plant J.* 55 504–513. 10.1111/j.1365-313X.2008.03519.x 18410479

[B6] Biasini M. Bienert S. Waterhouse A. Arnold K. Studer G. Schmidt T. (2014). SWISS-MODEL: modelling protein tertiary and quaternary structure using evolutionary information. *Nucleic Acid. Res.* 42 W252–W258. 10.1093/nar/gku340 24782522 PMC4086089

[B7] Blaudez D. Kohler A. Martin F. Sanders D. Chalot M. (2003). Poplar metal tolerance protein 1 confers zinc tolerance and is an oligomeric vacuolar zinc transporter with an essential leucine zipper motif. *Plant Cell* 15 2911–2928. 10.1105/tpc.017541 14630973 PMC282827

[B8] Boisson-Dernier A. Chabaud M. Garcia F. Bécard G. Rosenberg C. Barker D. G. (2001). Agrobacterium rhizogenes-transformed roots of *Medicago truncatula* for the study of nitrogen-fixing and endomycorrhizal symbiotic associations. *Mol. Plant Microbe Interact.* 14 695–700. 10.1094/MPMI.2001.14.6.695 11386364

[B9] Brewin N. J. (1991). Development of the legume root nodule. *Annu. Rev. Cell Biol.* 7 191–226. 10.1146/annurev.cb.07.110191.001203 1809347

[B10] Brito B. Palacios J. M. Hidalgo E. Imperial J. Ruíz-Argüeso T. (1994). Nickel availability to pea (*Pisum sativum* L.) plants limits hydrogenase activity of *Rhizobium leguminosarum* bv. viciae bacteroids by affecting the processing of the hydrogenase structural subunits. *J. Bacteriol.* 176 5297–5303. 10.1128/jb.176.17.5297-5303.1994 8071205 PMC196714

[B11] Broadley M. R. White P. J. Hammond J. P. Zelko I. Lux A. (2007). Zinc in plants. *New Phytol.* 173 677–702. 10.1111/j.1469-8137.2007.01996.x 17286818

[B12] Chen M. Shen X. Li D. Ma L. Dong J. Wang T. (2009). Identification and characterization of MtMTP1 a Zn transporter of CDF family, in the *Medicago truncatula*. *Plant Physiol. Biochem.* 47 1089–1094. 10.1016/j.plaphy.2009.08.006 19800247

[B13] Cheng H. P. Walker G. C. (1998). Succinoglycan is required for initiation and elongation of infection threads during nodulation of alfalfa by Rhizobium meliloti. *J. Bacteriol.* 180 5183–5191. 9748453 10.1128/jb.180.19.5183-5191.1998 PMC107556

[B14] Choi S. Hu Y.-M. Corkins M. E. Palmer A. E. Bird A. J. (2018). Zinc transporters belonging to the Cation Diffusion Facilitator (CDF) family have complementary roles in transporting zinc out of the cytosol. *PLoS Genet.* 14:e1007262. 10.1371/journal.pgen.1007262 29529046 PMC5864093

[B15] Coleman J. E. (1998). Zinc enzymes. *Curr. Opin. Chem. Biol.* 2 222–234. 10.1016/S1367-5931(98)80064-1 9667939

[B16] Desbrosses-Fonrouge A. G. Voigt K. Schroder A. Arrivault S. Thomine S. Kramer U. (2005). *Arabidopsis thaliana* MTP1 is a Zn transporter in the vacuolar membrane which mediates Zn detoxification and drives leaf Zn accumulation. *FEBS Lett.* 579 4165–4174. 10.1016/j.febslet.2005.06.046 16038907

[B17] DiDonato R. J. Roberts L. A. Sanderson T. Eisley R. B. Walker E. L. (2004). Arabidopsis Yellow Stripe-Like2 (YSL2) a metal-regulated gene encoding a plasma membrane transporter of nicotianamine-metal complexes. *Plant J.* 39 403–414. 10.1111/j.1365-313X.2004.02128.x 15255869

[B18] Downie J. A. (2014). Legume nodulation. *Curr. Biol.* 24 R184–R190. 10.1016/j.cub.2014.01.028 24602880

[B19] Ellis C. D. MacDiarmid C. W. Eide D. J. (2005). Heteromeric protein complexes mediate zinc transport into the secretory pathway of eukaryotic cells. *J. Biol. Chem.* 280 28811–28818. 10.1074/jbc.M505500200 15961382

[B20] Ellis C. D. Wang F. MacDiarmid C. W. Clark S. Lyons T. Eide D. J. (2004). Zinc and the Msc2 zinc transporter protein are required for endoplasmic reticulum function. *J. Cell Biol.* 166 325–335. 10.1083/jcb.200401157 15277543 PMC2172251

[B21] Eren E. Argüello J. M. (2004). Arabidopsis HMA2, a divalent heavy metal-transporting PIB-type ATPase, is involved in cytoplasmic Zn2 + homeostasis. *Plant Physiol.* 136 3712–3723. 10.1104/pp.104.046292 15475410 PMC527169

[B22] Eroglu S. Giehl R. F. H. Meier B. Takahashi M. Terada Y. Ignatyev K. (2017). Metal tolerance protein 8 mediates manganese homeostasis and iron reallocation during seeddevelopment and germination. *Plant Physiol.* 174 1633–1647. 10.1104/pp.16.01646 28461400 PMC5490884

[B23] Eroglu S. Meier B. von Wiren N. Peiter E. (2015). The vacuolar manganese transporter MTP8 determines tolerance to Fe deficiency-induced chlorosis in Arabidopsis. *Plant Physiol.* 170 1030–1045. 10.1104/pp.15.01194 26668333 PMC4734556

[B24] Gage D. J. (2002). Analysis of infection thread development using Gfp- and DsRed-expressing *Sinorhizobium meliloti*. *J. Bacteriol.* 184 7042–7046. 10.1128/JB.184.24.7042-7046.2002 12446653 PMC135452

[B25] González-Guerrero M. Matthiadis A. Sáez Á Long T. A. (2014). Fixating on metals: new insights into the role of metals in nodulation and symbiotic nitrogen fixation. *Front. Plant Sci* 5:45. 10.3389/fpls.2014.00045 24592271 PMC3923141

[B26] González-Guerrero M. Raimunda D. Cheng X. Argüello J. M. (2010). Distinct functional roles of homologous Cu + efflux ATPases in *Pseudomonas aeruginosa*. *Mol. Microbiol.* 78 1246–1258. 10.1111/j.1365-2958.2010.07402.x 21091508

[B27] González-Guerrero M. V. E. Sáez Á Tejada-Jiménez M. (2016). Transition metal transport in plants and associated endosymbionts. Arbuscular mycorrhizal fungi and rhizobia. *Front. Plant Sci.* 7:1088. 10.3389/fpls.2016.01088 27524990 PMC4965479

[B28] Hakoyama T. Niimi K. Yamamoto T. Isobe S. Sato S. Nakamura Y. (2012). The integral membrane protein SEN1 is required for symbiotic nitrogen fixation in *Lotus japonicus* nodules. *Plant Cell Physiol.* 53 225–236. 10.1093/pcp/pcr167 22123791

[B29] Hardy R. W. Holsten R. D. Jackson E. K. Burns R. C. (1968). The acetylene-ethylene assay for n(2) fixation: laboratory and field evaluation. *Plant Physiol.* 43 1185–1207. 10.1104/pp.43.8.1185 16656902 PMC1086994

[B30] Hussain D. Haydon M. J. Wang Y. Wong E. Sherson S. M. Young J. (2004). P-Type ATPase heavy metal transporters with roles in essential zinc homeostasis in Arabidopsis. *Plant Cell* 16 1327–1339. 10.1105/tpc.020487 15100400 PMC423219

[B31] Ibrikci H. Moraghan J. T. (1993). Differential responses of soybean and dry bean to zinc deficiency. *J. Plant Nutr.* 16 1791–1805. 10.1080/01904169309364650

[B32] Ishimaru Y. Suzuki M. Kobayashi T. Takahashi M. Nakanishi H. Mori S. (2005). OsZIP4, a novel zinc-regulated zinc transporter in rice. *J. Exp. Bot.* 56 3207–3214. 10.1093/jxb/eri317 16263903

[B33] Jones D. Taylo W. Thronton J. (1992). The rapid generation of mutation data matrices from protein sequences. *Comput. Appl. Biosci.* 8 275–282. 10.1093/bioinformatics/8.3.275 1633570

[B34] Kereszt A. Mergaert P. Kondorosi E. (2011). Bacteroid development in legume nodules: evolution of mutual benefit or of sacrificial victims? *Mol. Plant Microbe Interact.* 24 1300–1309. 10.1094/MPMI-06-11-0152 21995798

[B35] Kondorosi E. Mergaert P. Kereszt A. (2013). A Paradigm for endosymbiotic life: cell differentiation of Rhizobium bacteria provoked by host plant factors. *Annu. Rev. Microbiol.* 67 611–628. 10.1146/annurev-micro-092412-155630 24024639

[B36] Korshunova Y. O. Eide D. Clark W. G. Guerinot M. L. Pakrasi H. B. (1999). The IRT1 protein from *Arabidopsis thaliana* is a metal transporter with a broad substrate range. *Plant Mol. Biol.* 40 37–44. 10.1023/A:1026438615520 10394943

[B37] Li L. Chen O. S. Ward D. M. Kaplan J. (2001). CCC1 is a transporter that mediates vacuolar iron storage in yeast. *J. Biol. Chem.* 276 29515–29519. 10.1074/jbc.M103944200 11390404

[B38] Limpens E. Ivanov S. van Esse W. Voets G. Fedorova E. Bisseling T. (2009). Medicago N2-fixing symbiosomes acquire the endocytic identity marker Rab7 but delay the acquisition of vacuolar identity. *Plant Cell* 21 2811–2828. 10.1105/tpc.108.064410 19734435 PMC2768938

[B39] Livak K. J. Schmittgen T. D. (2001). Analysis of relative gene expression data using Real-Time Quantitative PCR and the 2-ΔΔCT method. *Methods* 25 402–408. 10.1006/meth.2001.1262 11846609

[B40] Lu M. Chai J. Fu D. (2009). Structural basis for autoregulation of the zinc transporter YiiP. *Nat. Struct. Mol. Biol.* 16 1063–1067. 10.1038/nsmb.1662 19749753 PMC2758918

[B41] Lu M. Fu D. (2007). Structure of the zinc transporter YiiP. *Science* 317 1746–1748. 10.1126/science.1143748 17717154

[B42] MacDiarmid C. W. Gaither L. Eide D. (2000). Zinc transporters that regulate vacuolar zinc storage in *Saccharomyces cerevisiae*. *EMBO J.* 19 2845–2855. 10.1093/emboj/19.12.2845 10856230 PMC203372

[B43] Marschner P. (2012). *Mineral Nutrition of Higher Plants* 3rd Edn. Cambridge, MA: Academic Press.

[B44] Migocka M. Kosieradzka A. Papierniak A. Maciaszczyk-Dziubinska E. Posyniak E. Garbiec A. (2015). Two metal-tolerance proteins, MTP1 and MTP4, are involved in Zn homeostasis and Cd sequestration in cucumber cells. *J. Exp. Bot.* 66 1001–1015. 10.1093/jxb/eru459 25422498

[B45] Montiel J. Szücs A. Boboescu I. Z. Gherman V. D. Kondorosi É Kereszt A. (2016). Terminal bacteroid differentiation is associated with variable morphological changes in legume species belonging to the inverted repeat-lacking clade. *Mol. Plant Microbe Interact.* 29 210–219. 10.1094/MPMI-09-15-0213-R 26713350

[B46] Morel M. Crouzet J. Gravot A. Auroy P. Leonhardt N. Vavasseur A. (2009). AtHMA3, a P1B-ATPase allowing Cd/Zn/Co/Pb vacuolar storage in Arabidopsis. *Plant Physiol.* 149 894–904. 10.1104/pp.108.130294 19036834 PMC2633814

[B47] Nakagawa T. Kurose T. Hino T. Tanaka K. Kawamukai M. Niwa Y. (2007). Development of series of gateway binary vectors, pGWBs, for realizing efficient construction of fusion genes for plant transformation. *J. Biosci. Bioeng.* 104 34–41. 10.1263/jbb.104.34 17697981

[B48] O’Hara G. W. (2001). Nutritional constraints on root nodule bacteria affecting symbiotic nitrogen fixation: a review. *Aust. J. Exp. Agric.* 41 417–433. 10.1071/EA00087

[B49] Oldroyd G. E. D. (2013). Speak, friend, and enter: signalling systems that promote beneficial symbiotic associations in plants. *Nat. Rev. Microbiol.* 11 252–263. 10.1038/nrmicro2990 23493145

[B50] Olsen L. I. Palmgren M. G. (2014). Many rivers to cross: the journey of zinc from soil to seed. *Front. Plant Sci.* 5:30. 10.3389/fpls.2014.00030 24575104 PMC3921580

[B51] Pedas P. Husted S. (2009). Zinc transport mediated by barley ZIP proteins are induced by low pH. *Plant Signal. Behav.* 4 842–845. 10.4161/psb.4.9.9375 19847115 PMC2802790

[B52] Podar D. Scherer J. Noordally Z. Herzyk P. Nies D. Sanders D. (2012). Metal selectivity determinants in a family of transition metal transporters. *J. Biol. Chem.* 287 3185–3196. 10.1074/jbc.M111.305649 22139846 PMC3270973

[B53] Portnoy M. E. Liu X. F. Culotta V. C. (2000). *Saccharomyces cerevisiae* expresses three functionally distinct homologues of the nramp family of metal transporters. *Mol. Cell Biol.* 20 7893–7902. 10.1128/MCB.20.21.7893-7902.2000 11027260 PMC86400

[B54] Ricachenevsky F. Menguer P. Sperotto R. Williams L. Fett J. (2013). Roles of plant metal tolerance proteins (MTP) in metal storage and potential use in biofortification strategies. *Front. Plant Sci.* 4:144. 10.3389/fpls.2013.00144 23717323 PMC3653063

[B55] Roux B. Rodde N. Jardinaud M.-F. Timmers T. Sauviac L. Cottret L. (2014). An integrated analysis of plant and bacterial gene expression in symbiotic root nodules using laser-capture microdissection coupled to RNA sequencing. *Plant J.* 77 817–837. 10.1111/tpj.12442 24483147

[B56] Rubio L. M. Ludden P. W. (2005). Maturation of nitrogenase: a biochemical puzzle. *J. Bacteriol.* 187 405–414. 10.1128/JB.187.2.405-414.2005 15629911 PMC543557

[B57] Saier M. H. Reddy V. S. Tamang D. G. Västermark Å. (2014). The transporter classification database. *Nucleic Acids Res* 42 D251–D258. 10.1093/nar/gkt1097 24225317 PMC3964967

[B58] Saitou N. Nei M. (1987). The neighbor-joining method: a new method for reconstructing phylogenetic trees. *Mol. Biol. Evol.* 4 406–425. 3447015 10.1093/oxfordjournals.molbev.a040454

[B59] Schiestl R. H. Gietz R. D. (1989). High efficiency transformation of intact yeast cells using single stranded nucleic acids as a carrier. *Curr. Genet.* 16 339–346. 10.1007/BF00340712 2692852

[B60] Sherman F. Fink G. R. Hicks J. B. (1981). *Methods in Yeast Genetics: Laboratory Manual.* New York, NY: Cold Spring Harbor Laboratory.

[B61] Sievers F. Wilm A. Dineen D. Gibson T. J. Karplus K. Li W. (2011). Fast, scalable generation of high-quality protein multiple sequence alignments using Clustal Omega. *Int Mol. Syst. Biol.* 7:539. 10.1038/msb.2011.75 21988835 PMC3261699

[B62] Sinclair S. A. Krämer U. (2012). The zinc homeostasis network of land plants. *Biochim. Biophys. Acta* 1823 1553–1567. 10.1016/j.bbamcr.2012.05.016 22626733

[B63] Stonoha-Arther C. Wang D. (2018). Tough love: accomodating intracellular bacteria through directed secretion of antimicrobial peptides during the nitrogen-fixing symbiosis. *Curr. Opin. Plant Biol.* 44 155–163. 10.1016/j.pbi.2018.04.017 29778978

[B64] Suzuki T. Ishihara K. Migaki H. Ishihara K. Nagao M. Yamaguchi-Iwai Y. (2005). Two different zinc transport complexes of Cation Diffusion Facilitator proteins localized in the secretory pathway operate to activate alkaline phosphatases in vertebrate cells. *J. Biol. Chem.* 280 30956–30962. 10.1074/jbc.M506902200 15994300

[B65] Timmers A. C. J. Soupène E. Auriac M. C. de Billy F. Vasse J. Boistard P. (2000). Saprophytic intracellular rhizobia in alfalfa nodules. *Mol. Plant Microbe Interact.* 13 1204–1213. 10.1094/MPMI.2000.13.11.1204 11059487

[B66] Udvardi M. Poole P. S. (2013). Transport and metabolism in legume-rhizobia symbioses. *Annu. Rev. Plant Biol.* 64 781–805. 10.1146/annurev-arplant-050312-120235 23451778

[B67] Van de Velde W. Zehirov G. Szatmari A. Debreczeny M. Ishihara H. Kevei Z. (2010). Plant peptides govern terminal differentiation of bacteria in symbiosis. *Science* 327 1122–1126. 10.1126/science.1184057 20185722

[B68] Vasse J. de Billy F. Camut S. Truchet G. (1990). Correlation between ultrastructural differentiation of bacteroids and nitrogen fixation in alfalfa nodules. *J. Bacteriol.* 172 4295–4306. 10.1128/jb.172.8.4295-4306.1990 2376562 PMC213254

[B69] Vernoud V. Journet E. P. Barker D. G. (1999). MtENOD20 a Nod factor-inducible molecular marker for root cortical cell activation. *Mol. Plant Microbe Interact.* 12 604–614. 10.1094/MPMI.1999.12.7.604

[B70] Waters B. M. Chu H.-H. DiDonato R. J. Roberts L. A. Eisley R. B. Lahner B. (2006). Mutations in Arabidopsis Yellow Stripe-Like1 and Yellow Stripe-Like3 reveal their roles in metal ion homeostasis and loading of metal ions in seeds. *Plant Physiol.* 141 1446–1458. 10.1104/pp.106.082586 16815956 PMC1533956

[B71] Waters B. M. Grusak M. A. (2008). Quantitative trait locus mapping for seed mineral concentrations in two *Arabidopsis thaliana* recombinant inbred populations. *New Phytol.* 179 1033–1047. 10.1111/j.1469-8137.2008.02544.x 18631293

[B72] Wei Y. Fu D. (2006). Binding and transport of metal ions at the dimer interface of the *Escherichia coli* metal transporter YiiP. *J. Biol. Chem.* 281 23492–23502. 10.1074/jbc.M602254200 16790427

[B73] Wei Y. Li H. Fu D. (2004). Oligomeric state of the *Escherichia coli* metal transporter YiiP. *J. Biol. Chem.* 279 39251–39259. 10.1074/jbc.M407044200 15258151

[B74] Xi J. L. Chen Y. Nakashima J. Wang S. M. Chen R. (2013). *Medicago truncatula* esn1 defines a genetic locus involved in nodule senescence and symbiotic nitrogen fixation. *Mol. Plant Microbe Interact.* 26 893–902. 10.1094/MPMI-02-13-0043-R 23634841

[B75] Xiao T. T. Schilderink S. Moling S. Deinum E. E. Kondorosi E. Franssen H. (2014). Fate map of *Medicago truncatula* root nodules. *Development* 141 3517–3528. 10.1242/dev.110775 25183870

