# Supplementary material for: MtMTP2-Facilitated Zinc Transport Into Intracellular Compartments Is Essential for Nodule Development in Medicago truncatula
Source: Front Plant Sci. 2018 Jul 10;9:990. doi: 10.3389/fpls.2018.00990 (PMC6048390; doi:10.3389/fpls.2018.00990)
Supplement: Supplementary file 1 [file Image_1.PDF]

Supporting Information Figure S1. Expression of *Medicago truncatula* MTP family members in different plant organs. A Data obtained from the Symbimics database (https://iant.toulouse.inra.fr/symbimics/). **B** Data obtained from the *Medicago* Gene Expression Atlas (https://mtgea.noble.org/v3/).

Supporting Information Figure S2. Expression of *MtMTP2* in *M. truncatula* nodules by laser-capture microdissection coupled to RNA sequencing. Data were obtained from Symbimics database (https://iant.toulouse.inra.fr/symbimics/). ZI, Zone I; ZIIp, Zone II proximal; ZIId, Zone II distal; IZ, interzone; ZIII, Zone III.

Supporting Information Figure S3. **Localization of *pMtMTP2::MtMTP2-HA* in *Medicago truncatula*.** Cross section of a 28-dpi *M. truncatula* nodule transiently expressing the *pMtMTP2::MtMTP2-HA* construct (red) and inoculated with a *Sinorhizobium meliloti* 20.11 pHC60 strain constitutively expressing GFP (green). DNA was stained using 4'-6-diamino-phenylindole (DAPI) (blue). Top left panel, DAPI channel; top right panel, GFP channel; lower left panel, Alexa 594 channel; lower right panel, overlay of the three previous channels with transillumination. Bars: 100 µm.

Supporting Information Figure S4. Autofluorescence control for Alexa 594 emission. A. Inmunolocalization of *pMtMTP2::MtMTP2-HA* in a cross section of 28-dpi *M. truncatula* nodules transiently expressing *pMtMTP2::MtMTP2-HA* construct, inoculated with *S. meliloti* pHC60 constitutively expressing GFP (green). These nodules were not incubated with the mouse anti-HA primary antibody to be used as a negative control. DNA was stained using DAPI (blue). Top left panel, DAPI channel; top right panel, GFP channel; lower left panel, Alexa 594 channel; lower right panel, transillumination image. Bars: 100 µm.

Supporting Information Figure S5. Autoflorescence control for *pMtMTP2::MtMTP2-GFP* emission. Cross section of non-transformed 28-dpi *M. truncatula* nodules, inoculated with *S. meliloti* 1021 expressing constitutively DsRED (red). DNA was stained using DAPI (blue). Top left panel, DAPI channel; top right panel, GFP channel; lower left panel, DsRed channel; lower right panel, transillumination image. Bars: 100 µm

Supporting Information Figure S6. Transmission electron microscopy (TEM) image control for *pMtMTP2::MtMTP2-HA* immunolocalization. Transmission electron microscopy (TEM) image was obtained from an infected cell of a 28-dpi *M. truncatula* nodule transiently expressing *pMtMTP2::MtMTP2-HA* and inoculated with *S. meliloti* 2011 without incubation with the mouse anti-HA primary antibody. Bar: 2 µm.

Supporting Information Figure S7. *MtMTP2* mutants grown under non-symbiotic conditions did not show an altered phenotype regardless of zinc concentration in the nutrient solution. A. From the left to the right, representative plants of *M. truncatula* WT, *mtp2-1* and *mtp2-2* N-fertilized plants watered with a nutrient solution containing 0, 0.38, or 38 µM $ZnSO_4$. Bar: 3 cm **B.** Dry weight of shoots (black) and roots (white). Data represent the mean ± SD of three experiments pooling, at least, ten independent plants (n=10).

Supporting Information Figure S8. Time-course of nodule development in *M. truncatula* WT, *mtp2-1* and *mtp2-2* plants. **A**. Time-course of nodule development in WT and *MtMTP2* mutant lines. Bars: 50 µm. **B**. Nodulation kinetics in WT and *MtMTP2* mutant lines. Data are the mean ± SE from ten plants.

Supporting Information Figure S9. Nitrogenase activity of 28 dpi nodules standardized to nodule number. Acetylene reduction was measured in duplicate from three sets of four pooled plants. Data are the mean ± SD.

Supporting Information Figure S10. *Medicago truncatula MTP2* mutation impairs nitrogen fixation. A. Representative WT, *mtp2-1* and *mtp2-2* plants. +/+ indicates *mtp2* segregants with two wild-type copies of *MtMTP2,* while -/- indicate that both copies carry the insertion of *Tnt1.* Bar: 1 cm. **B**. Representative nodules of WT, *mtp2-1* and *mtp2-2* plants. +/+ indicates *mtp2* segregants with two wild-type copies of *MtMTP2,* while -/- indicate that both copies carry the insertion of *Tnt1.* Bars: 500 µm. **C.** Dry weight of shoots (black) and roots (white). Data are the mean ± SD of, at least, ten plants. **D**. Nitrogenase activity of 28-dpi nodules. Acetylene reduction was measured in duplicate from three sets of four pooled plants. Data are the mean ± SD. Values with different letters are significantly different (Tukey's HSD, $p < 0.05$).

Supporting Information Figure S11. *M. truncatula* WT and *mtp2-2* mutant plants growing under a zinc gradient. A. Representative plants of WT and *mtp2-2* growing in a Zn gradient (0, 3.8 and 38 µM $ZnSO_4$). Bar: 1 cm **B**. Dry weight of shoots (black) and roots (white) in each Zn condition. Data are the mean ± SD of, at least, ten plants. **C.** Nitrogenase activity of 28-dpi nodules developed in each zinc condition. Acetylene reduction was measured in duplicate from two sets of four pooled plants. Values with different letters are significantly different (Student's t-test, $p < 0.05$). Data are the mean ± SD.

SUPPORTING INFORMATION FIGURE S1

A

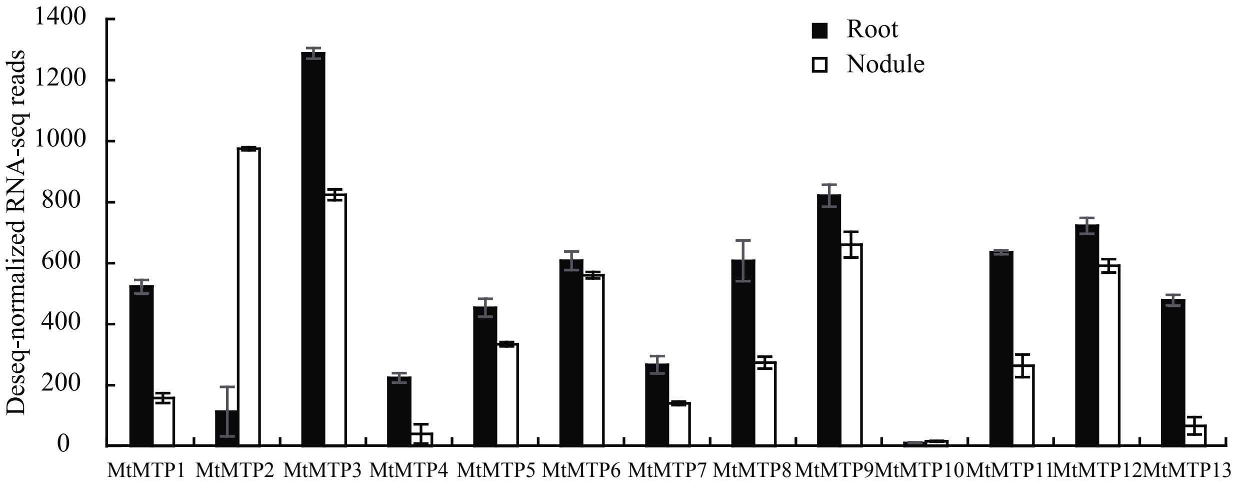

B

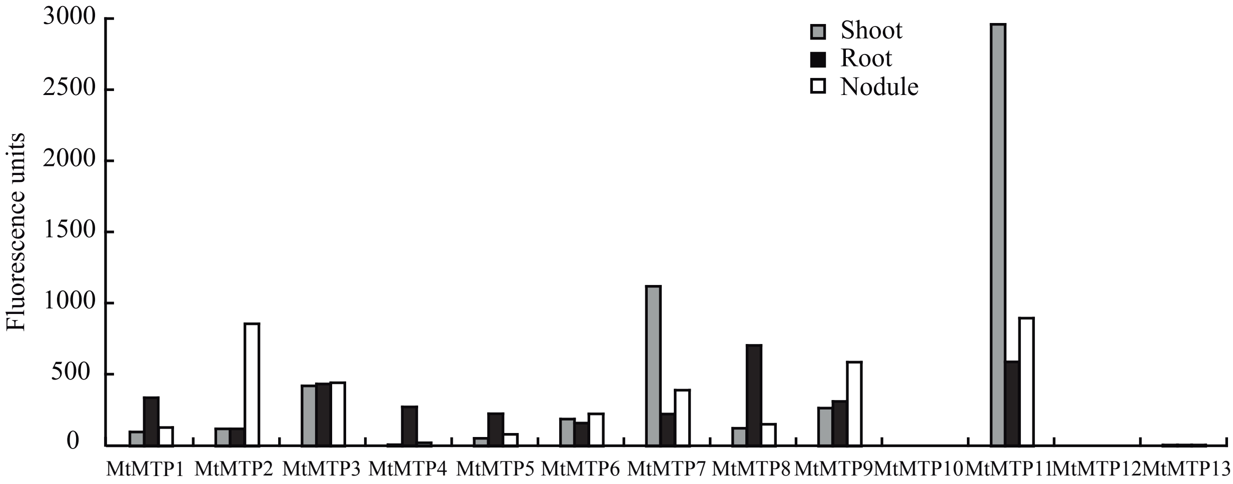

SUPPORTING INFORMATION FIGURE S2

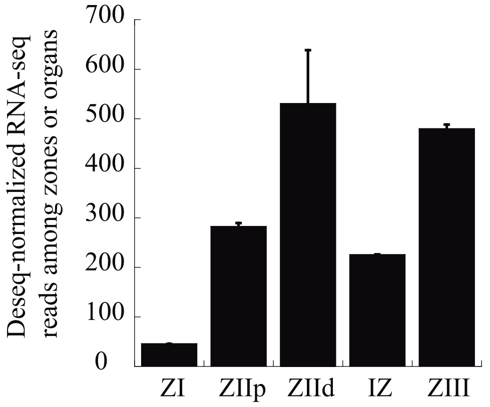

SUPPORTING INFORMATION FIGURE S3

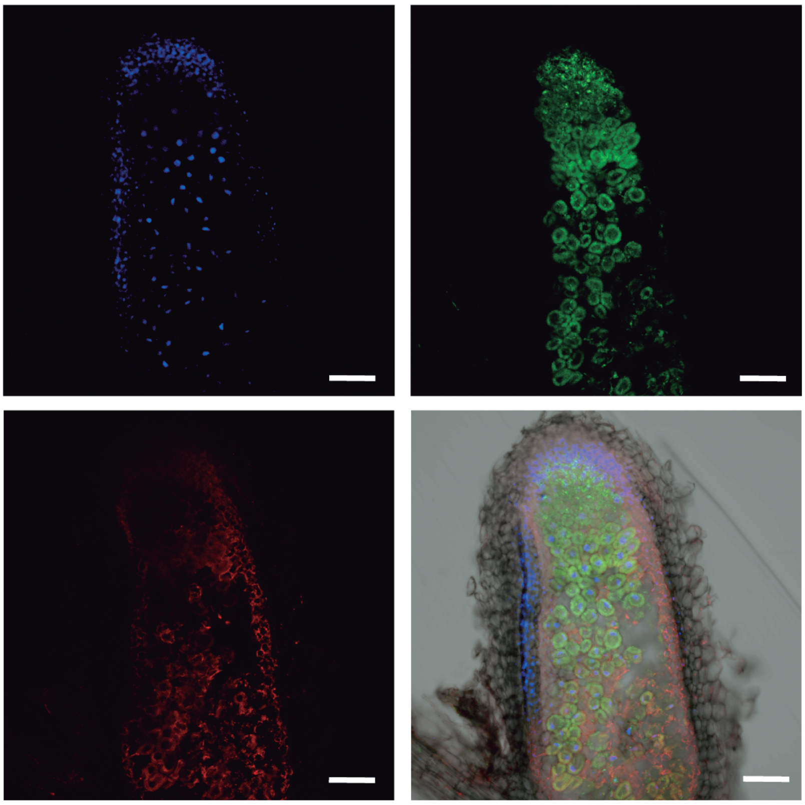

SUPPORTING INFORMATION FIGURE S4

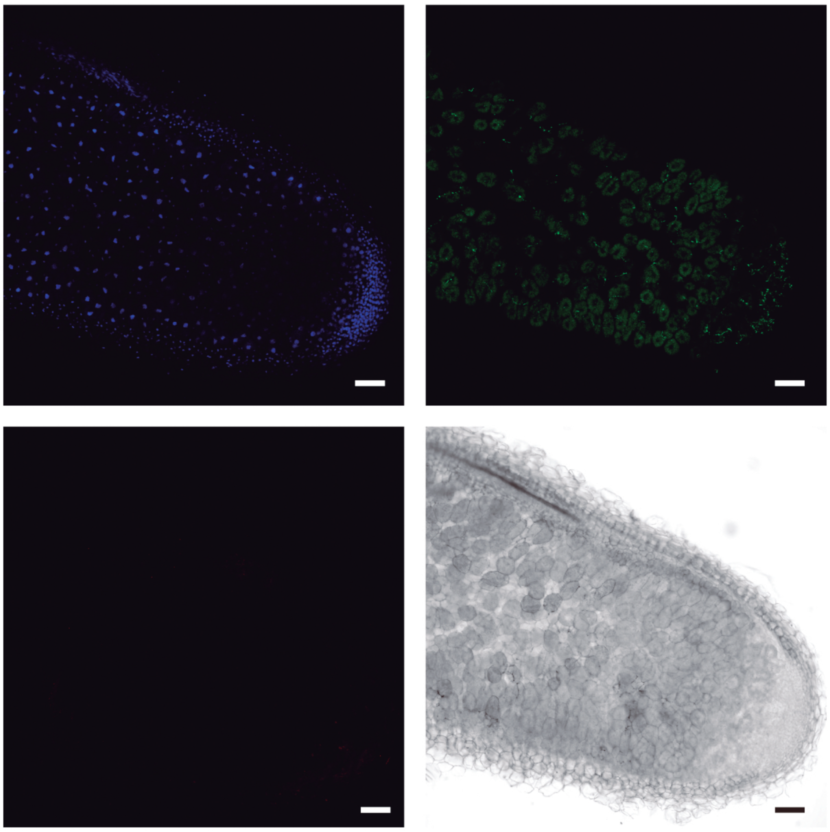

SUPPORTING INFORMATION FIGURE S5

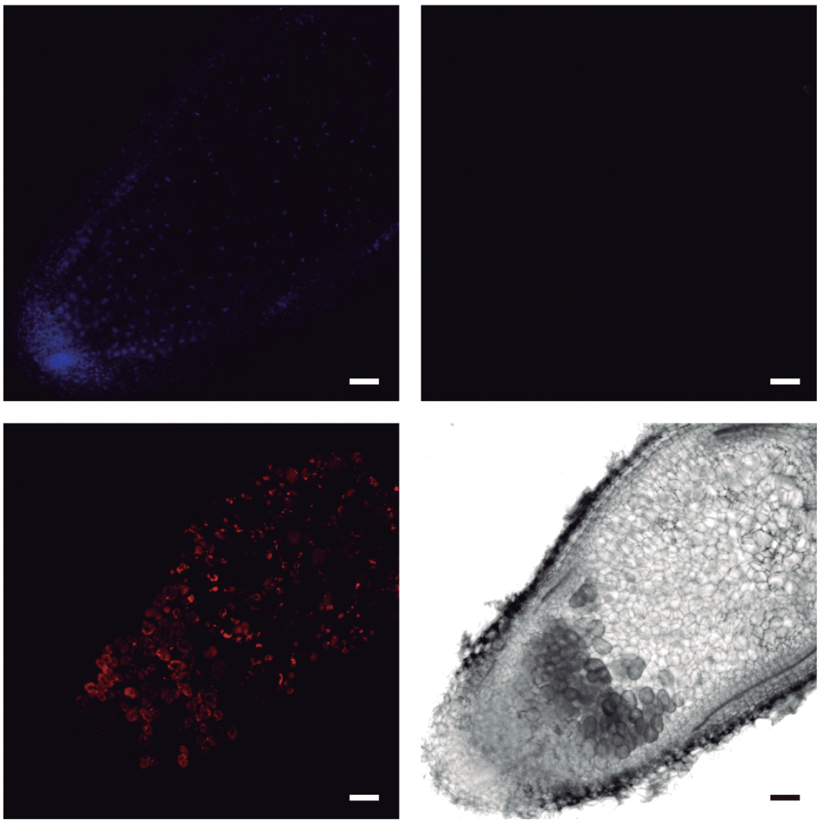

SUPPORTING INFORMATION FIGURE S6

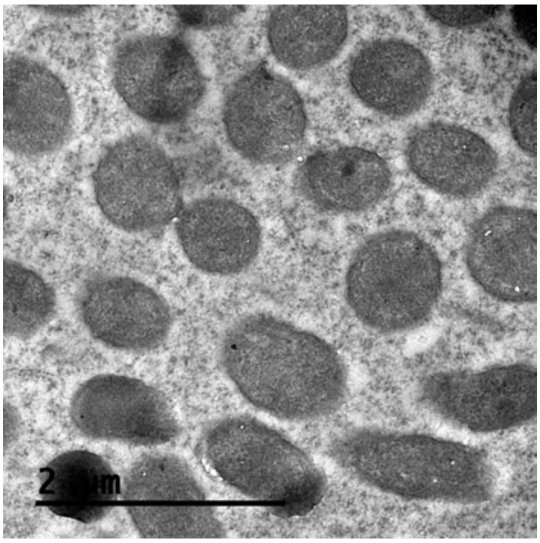

SUPPORTING INFORMATION FIGURE S7

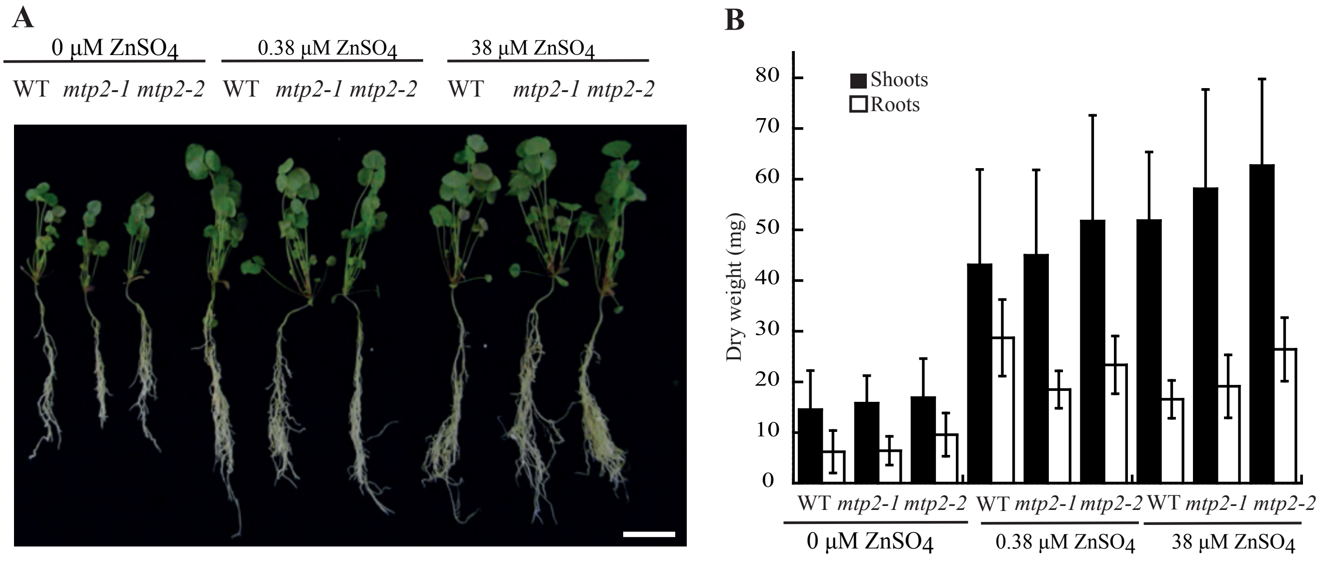

SUPPORTING INFORMATION FIGURE S8

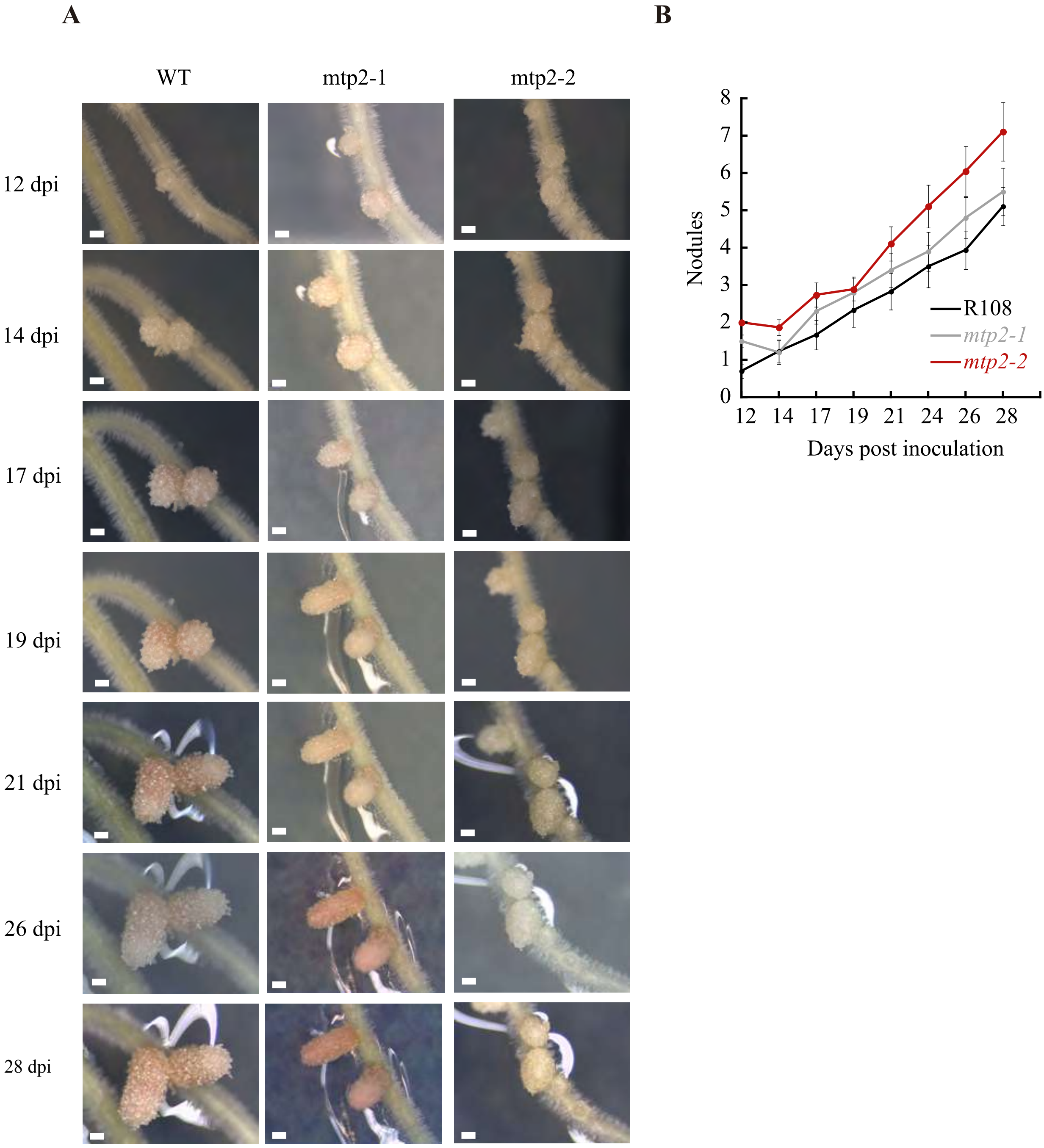

SUPPORTING INFORMATION FIGURE S9

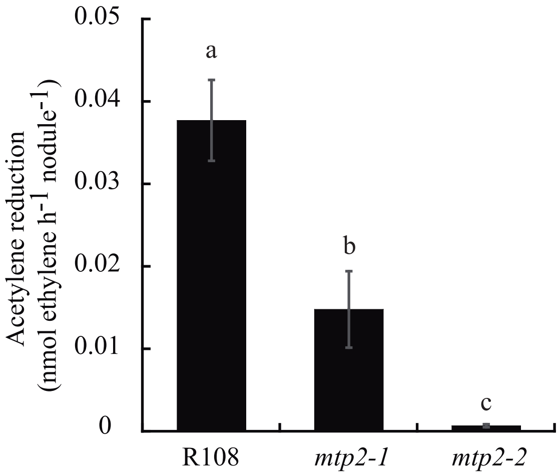

SUPPORTING INFORMATION FIGURE S10

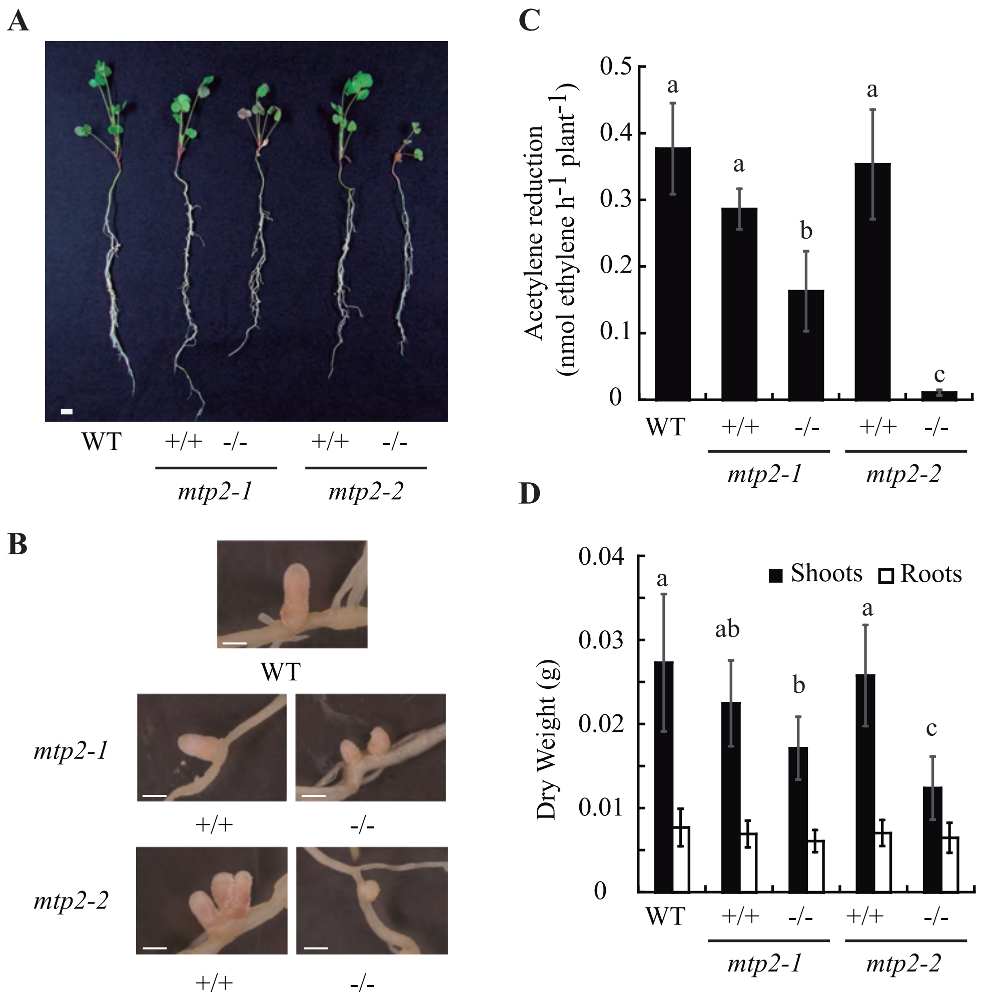

SUPPORTING INFORMATION FIGURE S11

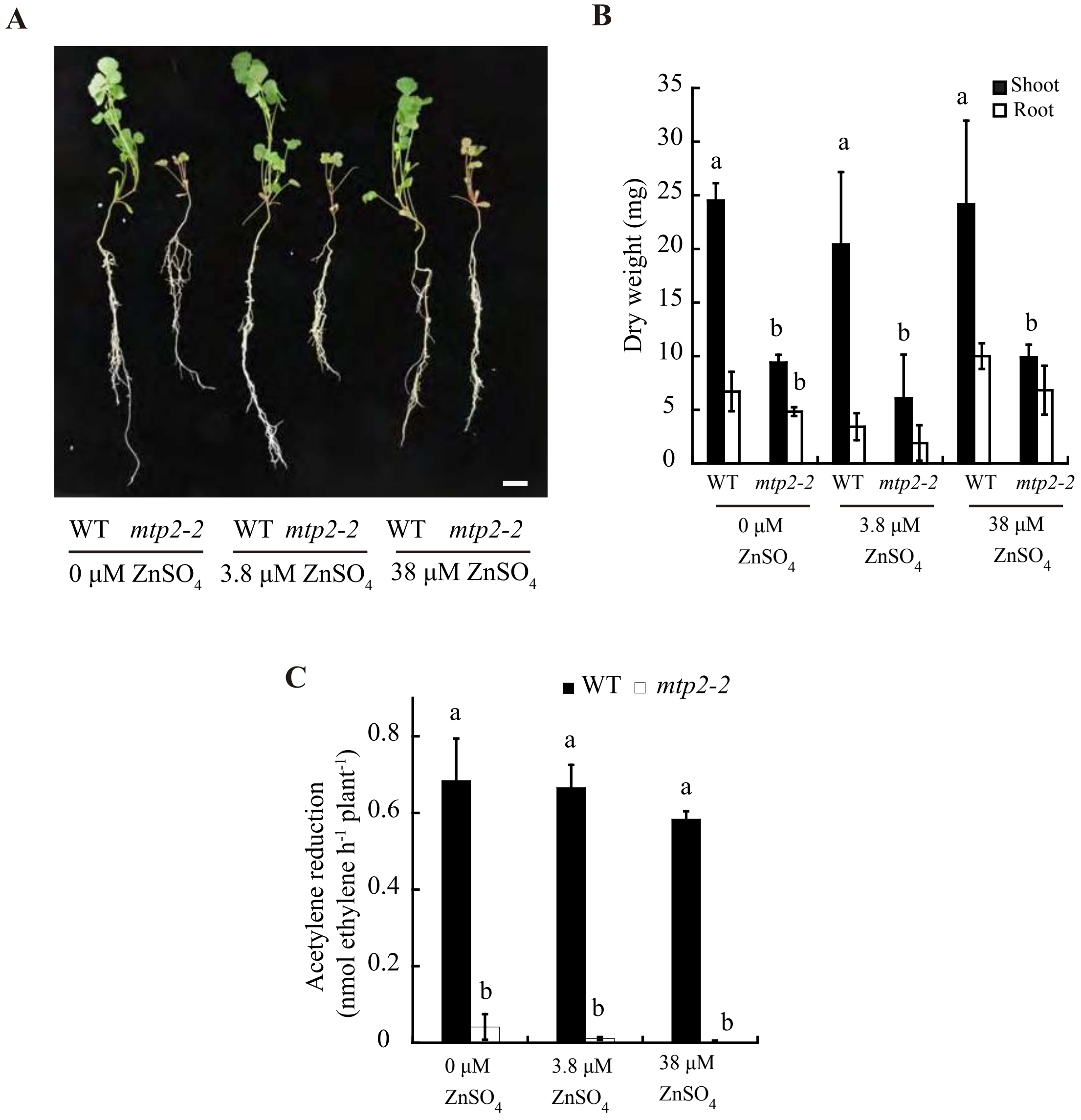

Supporting Information Table S1. Primers used in this study.

Name	Sequence	Use
5 MtUb v4qF	ATTCTTCACATGCGGCGATTTAC	Quantitative expression of MtUbiquitin carboxyl-terminal hydrolase
3 MtUb v4qR	TTTCTCATTTGCTTTTGGTGTTGG	Quantitative expression of MtUbiquitin carboxyl-terminal hydrolase
5 MtMTP2 q996 F	TGGAGAGAACGCCAAGTGAG	Quantitative expression of MtMTP2
3 MtMTP2 q1105 R	GCCAGGTTCAGCCACTACAT	Quantitative expression of MtMTP2
Tnt1-3	TGTAGCACCGAGATACGGTAATTAACAAGA	Genotyping mutant lines
Tnt1-4	TCCTTGTTGGATTGGTAGCCAACTTTGTTG	Genotyping mutant lines
5 MtMTP2 NF11171 -538 F	AATGTCTCAATTGCGTCTTC	Genotyping NF11171 mutant line
3 MtMTP2 NF11171 336 R	GATTATACCCGAAAGATTGATG	Genotyping NF11171 mutant line
5 MtMTP2 Pst1 pDR196 F	TTTTTCTGCAGATGAAGCAAATGGAACACGAG	MtMTP2 cDNA cloning for yeast complementation and genotyping NF18305 mutant line
3 MtMTP2 Xho1 pDR196 R	TTTTTCTCGAGTTACTCAATTTGTATGGTTACATGT TG	MtMTP2 cDNA cloning for yeast complementation and genotyping NF18305 mutant line
5 MtMTP2 XbaI pMBV	CTTTTTACAACAAATATAAAAACAAGATCTCGACT CTAGAATGACTTTTCTAGAATGGTTTA	MtMTP2 cDNA cloning for yeast complementation
3 MtMTP2 BamHI pAMBv	CGATAAGCTTGATATCGAATTCCTGCAGCCCGGGG GATCCTTACTCAATTTGTATGGTTAC	MtMTP2 cDNA cloning for yeast complementation
5 MtMTP2 pGW F	GGGGACAAGTTTGTACAAAAAAGCAGGCTTTCTGA AAGAATCCCTATAC	MtMTP2 promoter cloning into pGWB3
3 MtMTP2 pGW R	GGGGACCACTTTGTACAAGAAAGCTGGGTAGCCTA TAAAAACAAATAAACC	MtMTP2 promoter cloning into pGWB3
5 MtMTP2 -1961Kb GW F	GGGGACAAGTTTGTACAAAAAAGCAGGCTTTGCTT TTTCCTAACAGCAG	MtMTP2 genomic region cloning into pGWB4 and pGWB13
3 MtMTP2 full GW R	GGGGACCACTTTGTACAAGAAAGCTGGGTACTCAA TTTGTATGGTTACATG	MtMTP2 genomic region cloning into pGWB4 and pGWB13
3 MtCystProt qPCR F	TTTTCTGCTTGGGATTGTGG	Quantitative expression of MtCysteine proteinase
5 MtCystProt qPCR R	GTGATTCATCCACCGCTCAT	Quantitative expression of MtCysteine proteinase
3 MtChit2 qPCR F	CATTTCATCAACCCATGTTGG	Quantitative expression of MtChitinase 2
5 MtChit2 qPCR R	TTGTTGGATTCAGCTCCAATG	Quantitative expression of MtChitinase 2
